# Influence of fluorine substitution in naphthalenediimide–bithiophene (NDI–2T)-based n-type conjugated polymers for organic electrochemical transistors and glucose biosensors

**DOI:** 10.1039/d6ra03225g

**Published:** 2026-07-29

**Authors:** Xinnian Jiang, Xiandi Yang, Jiazheng Li, Wenhao Zuo, Zhi Li, Man Wang, Qiaogan Liao, Junyu Li, Tiedong Cheng, Ping Zhang, Yanxi Zhang, Gang Ye

**Affiliations:** a School of Electrical Engineering and Automation, Jiangxi University of Science and Technology Ganzhou Jiangxi 341000 China p.zhang@jxust.edu.cn; b Institute of Flexible Electronics (IFE, Future Technologies), Xiamen University Xiamen 361005 China ifeyxzhang@xmu.edu.cn; c Ministry of Education Key Laboratory for the Green Preparation and Application of Functional Materials, Hubei Key Laboratory of Polymer Materials, School of Materials Science and Engineering, Hubei University Youyi Road 368 Wuhan 430062 P. R. China g.ye0612@hubu.edu.cn; d School of Materials Science and Engineering, Guilin University of Electronic Technology Guilin Guangxi 541004 China; e Sinopec Shanghai Research Institute of Petrochemical Technology Shanghai 201028 China; f Jiangxi Provincial Key Equipment Industry Technology Engineering Center for Microgrids Ganzhou Jiangxi 341000 China

## Abstract

Fluorine (F) substitution in organic semiconductors has been proven effective for enhancing the performance of organic photovoltaics (OPVs), organic field-effect transistors (OFETs) and organic thermoelectrics (OTEs). However, the effect of such substitution on conjugated polymers in organic electrochemical transistors (OECTs) has not been fully elucidated. Herein, two conjugated polymers (PNDI-2T and PNDI-2TF) with a naphthalenediimide (NDI)-bithiophene (2T) backbone, featuring amphipathic side chains and either bearing or lacking fluorine (F) substitution on the bithiophene unit, were designed and synthesized to investigate their effect on organic electrochemical transistors. By combining optical spectroscopy, density functional theory calculations, cyclic voltammetry, and water contact angle analysis, we reveal that fluorine substitution on the conjugated polymer, on one hand, increases the hydrophobicity, which impedes the ion penetration, on the other hand, reduces the LUMO energy level of the polymer. As a consequence, PNDI-2TF-based OECT devices exhibited lower geometry-normalized transconductance performance but with a significantly reduced *V*_th_ to 0.232 V compared to PNDI-2T-based ones (0.386 V). Atomic force microscopy and 2D grazing-incidence wide-angle X-ray scattering reveal that fluorine substitution reduces polymer crystallinity, leading to decreased electron mobility and consequently inferior OECT performance in PNDI-2TF. In addition, we fabricated complementary inverters by pairing the p-type polymer (Pg2T-TT) with the n-type polymers (PNDI-2T or PNDI-2TF), achieving maximum voltage gains of 28.3 and 22.9 V/V for PNDI-2T and PNDI-2TF, respectively, at a supply voltage of 0.7 V. Furthermore, glucose sensors employing N-type conjugated polymers (PDNI-2T or PDNI-2TF) as the active layer exhibited both comparable and remarkably high sensitivity toward glucose sensing, together with an outstanding linear response over a wide concentration range of 1 µM to 20 mM, surpassing the performance of most reported electrochemical glucose sensors. Overall, this work not only elucidates the influence of fluorination on NDI-based polymers, an insight crucial for validating the fluorine substitution strategy in developing high-performance n-type organic mixed ionic-electronic conductors, but also demonstrates the promising prospects of NDI-based polymers in biological applications.

## Abstract 2

Conjugated polymers PNDI-2T and PNDI-2TF, were synthesized to investigate the effect on OECT. We find that F on the polymer, lower *g*_m,norm_ performance but with a significantly reduced *V*_th_.

## Graphical abstract

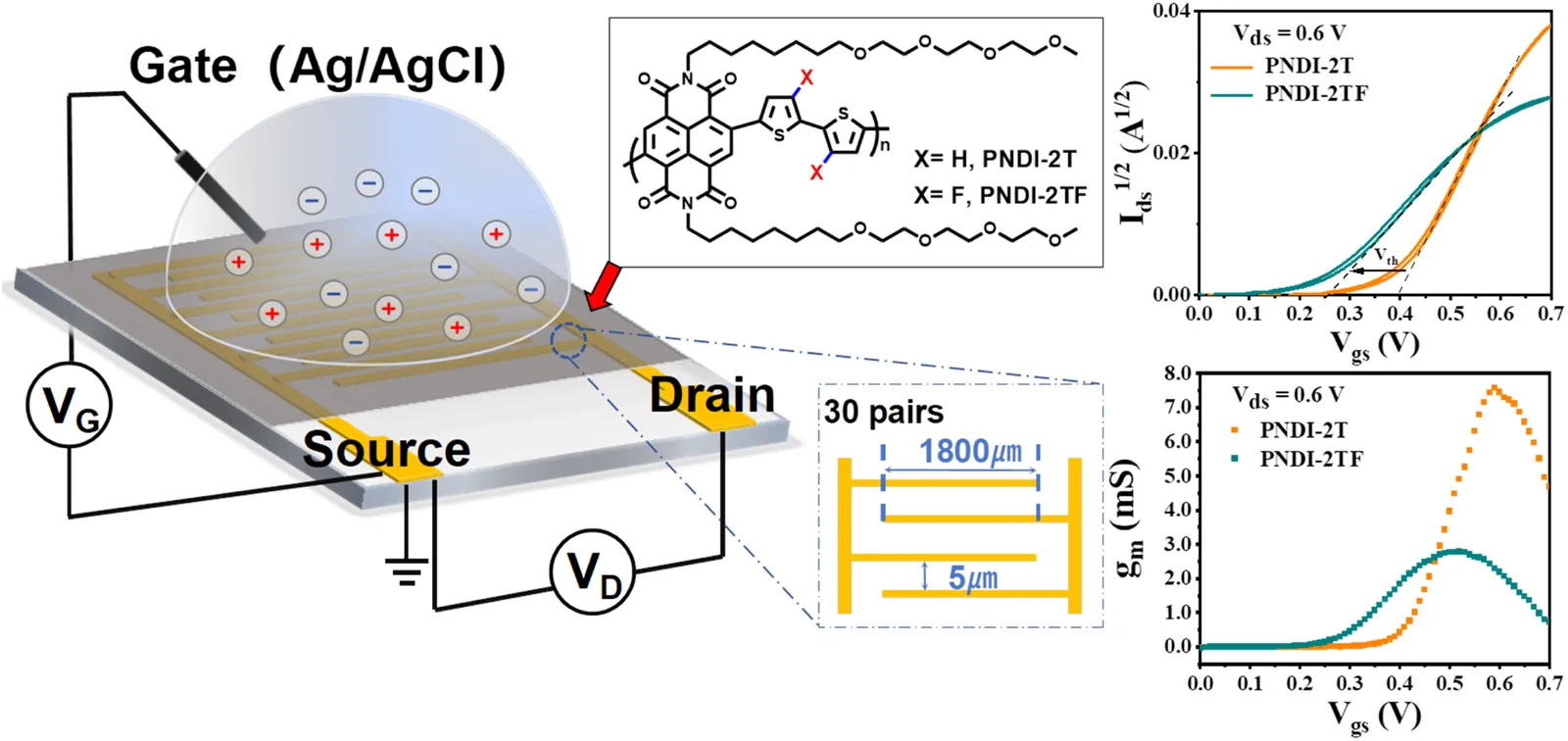

## 1 Introduction

Conjugated polymers that can efficiently facilitate both ionic and electronic transport simultaneously (mixed conduction) have drawn great attention recently as channel active materials for organic electrochemical transistors (OECTs),^1–5^ which have emerged as a promising class of devices for next-generation bioelectronics due to various advantageous features, such as low operating voltage (<1 V),^6,7^ signal amplification and transduction capabilities (transconductance >10 mS),^8^ favorable mechanical flexibility,^9,10^ scalable fabrication processes^11,12^ and great biocompatibility.^13–15^ These distinctive advantages have facilitated a wide array of bioelectronic applications, including wearable electronics,^16,17^ biological signal sensors,^18–23^ and neuromorphic computing devices.^24–27^ A typical OECT device consists of three-terminal configuration (source, drain, and gate) electrodes, where a conjugated polymer thin-film channel is patterned between two metal electrodes (drain and source). Specifically, the channel is in contact with an electrolyte where a gate electrode is immersed.^26^ The operation mechanism of OECTs involves injecting ions into the channel active materials *via* the gate bias (*V*_G_) and inducing the electrochemical doping of the active materials by electrolyte ions for charge compensation.^1,2^ Therefore, the conductance of the channel is probed by measuring the drain current (*I*_D_) when applying a bias between the drain and source (*V*_D_). The figure of merit to evaluate the performance of an OECT is the following equation:^28,29^1
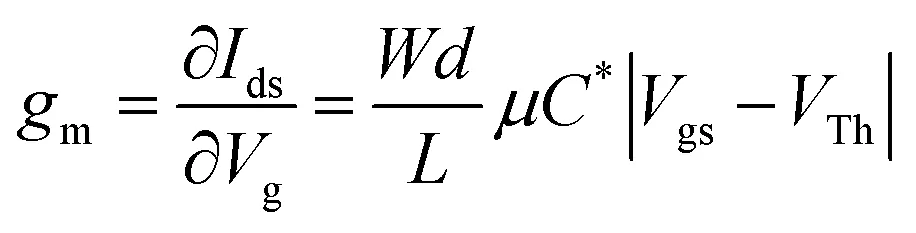
where *g*_m_ is the transconductance; *I*_ds_ is the drain current; *L*, *W*, *d* are the channel length, width, and film thickness, respectively; *µ* is the charge carrier mobility; C* is the volumetric capacitance, *V*_Th_ is the threshold voltage, and *V*_G_ is the applied gate voltage. Thus, the idea active materials for OECT require both high charge carrier mobility (*µ*) and ion uptake capability (C*).

Conventional conjugated polymers with high charge (hole/electron) mobilities for application in organic field-effect transistors (OFETs) cannot be directly used as active materials in OECTs, due to the closely packed polymer backbone chains and hydrophobic side chains that prevent efficient ion uptake and negatively affect ion transport in aqueous media.^30^ To overcome this bottleneck and facilitate ion-electron transport, hydrophilic oligo(ethylene glycol) (OEG) side chains have been widely used to replace the hydrophobic alkyl side chains on the conjugated polymer backbones.^31^ In such modification, hydrated ions and water molecules can penetrate the bulk of polymer films to enhance the ion mobility, resulting in a large number of high-performance p-type and n-type OECT materials like pg2T-T,^32^ p(g2T-TT),^33^ p(g2T2-g4T2),^34^ P-90,^30^ P-6O,^35^ f-BSeI2g-SVSCN,^21^ and PBTI2g-DTCN.^36^ However, several investigations have demonstrated that excessive water uptake can also lead to the negative swelling of polymer films, disrupting the interconnections between crystallites and adversely impacting the transport of electronic charges, resulting in a compromised OECTs performance.^37,38^ To achieve a balance between water uptake and electronic charge mobility, new synthetic strategies have been explored where the chemical structure is systematically altered to control the degree of hydration and limit the swelling of the channel material.^39,40^ The introduction of hydrophobic alkyl chain spacers between the conjugated backbone and OEG side chains has been demonstrated as a successful design strategy to minimize detrimental swelling and balance the mixed conduction properties of channel materials in aqueous electrolytes.^41,42^ Beyond side chain engineering, the polymer backbone modification is also the most effective method to enhance the performance of channel materials. Fluorine functionalization on the conjugated polymer backbone has been proven as a powerful method to build novel and high-performance conjugated polymers with improved charge mobility and reduced the low-lying lowest unoccupied molecular orbital (LUMO) energy levels. This approach has led to the successful development of excellent n-type polymers for high-performance OFETs, organic thermoelectrics (OTEs), and all-polymer solar cells (all-PSCs).^43^ Very recently, the fluorine substitution strategy has also been demonstrated in the development of excellent n-type polymers for high-performance OECTs.^44,45^

NDI-based n-type conjugated polymers are widely adopted as channel materials for n-type OECTs, owing to their intrinsic electron-accepting character, reversible electrochemical reduction, and tunable ion transport capability. The pioneering NDI-based conjugated polymer p(gNDI-gT2) developed by Giovannitti *et al.* exhibited outstanding n-type performance (µC* of 0.06 F cm^−1^ V^−1^ s^−1^) and excellent aqueous stability showing no degradation when tested for 2 hours under continuous cycling.^46^ Based on the success of p(gNDI-gT2), Inal *et al.* reported amphiphilic side-chain engineering and synthesized two polymers, p(C3-gNDI-gT2) and p(C6-gNDI-gT2), *via* precise modulation of hybrid alkyl/alkoxy side-chain lengths. Both polymers p(C3-gNDI-gT2) and p(C6-gNDI-gT2) exhibit enhanced µC* values of 0.13 and 0.16 F cm^−1^ V^−1^ s^−1^, together with improved operational stability.^47^ Subsequently, Giovannitti *et al.* employed random copolymerization strategy gradually replacing the alkyl side chains of benchmark polymer N2200 with ethylene glycol side chains to produce the organic mixed ionic-electronic conductors (OMIECs) P-75, P-90 and P-100, which delivered remarkable n-type performance (normalized *g*_m_: 0.141 S cm^−1^ for P-75, 0.210 S cm^−1^ for P-90 and 0.204 S cm^−1^ for P-100).^30^ Yue *et al.* adopted the fluorine substitution strategy and reported an NDI-based n-type OMIEC polymer, gNDI-FBT. This material outperformed its NDI-based polymer analogs in OECT devices, featuring a low threshold voltage of 0.19 V, a fast response time of 45.5 ms, and a high µC* values of 0.12 F cm^−1^ V^−1^ s^−1^.^44^

Motivated by the above advances, in this work, we integrate amphiphilic side-chain engineering with backbone fluorine functionalization to rationally design and synthesize two n-type conjugated polymers, PNDI-2T and PNDI-2TF. These polymers serve as active materials for OECTs, enabling a systematic investigation of how fluorinated donor functionalization modulates mixed ionic–electronic conduction and its consequent impact on inverter operation and glucose sensing performance. Both polymers exhibit n-type OECT behavior, delivering high geometry-normalized maximum transconductance values of 0.038 S cm^−1^ for PNDI-2T and 0.020 S cm^−1^ for PNDI-2TF, respectively. The reduced performance of PNDI-2TF is primarily attributed to the increased hydrophobicity induced by fluorine substitution, which raises the energy barrier for ion uptake. Nevertheless, backbone fluorination plays a decisive role by lowering the LUMO energy level, which in turn significantly reduces the threshold voltage (*V*_th_) of the n-type OECTs. Based on the above findings, we further demonstrate the practical applicability of these two n-type conjugated polymers. By integrating PNDI-2T or PNDI-2TF with the benchmark p-type material Pg2T-TT, complementary inverters were successfully constructed, achieving maximum voltage gains of 28.2 V/V and 23.0 V/V, respectively, at a low supply voltage of 0.7 V. In addition, leveraging the favorable mixed ionic–electronic conduction of both polymers, OEG side chains were utilized to enable physical adsorption of redox enzymes, yielding glucose sensors with high sensitivity and wide linear detection ranges. Overall, this work establishes backbone fluorination combined with amphiphilic side-chain engineering as a molecular design strategy to balance mixed ionic–electronic transport and electronic energetics in n-type conjugated polymers, thereby enabling effective bioelectronic circuits and high-performance biochemical sensing.

## 2 Results and discussion

### 2.1. Polymer synthesis and characterization

**Scheme 1:**
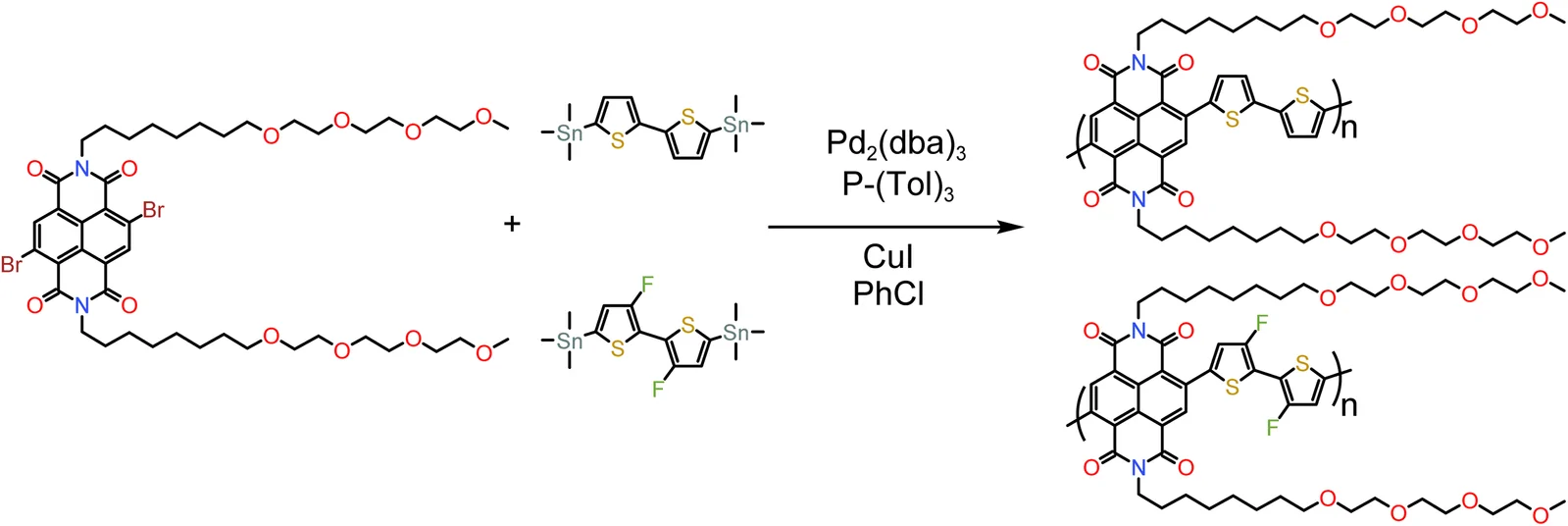
Synthetic route to the NDI-based copolymers PNDI-2T and PNDI-2TF.

Scheme 1 displays the synthetic route and corresponding structures of the investigated NDI-based copolymers PNDI-2T and PNDI-2TF. The copolymers were synthesized by a typically palladium-catalysted Stille polycondensation of symmetrical dibromo-NDI monomer and distannyl-thiophene based monomers after refluxing the degassed polymerization mixture overnight. The resulting copolymers were purified to remove low-molecular-weight fractions and impurities by continuous extraction with hot methanol followed by hexane in a Soxhlet extractor. Finally the polymers were extracted with chloroform, precipitated in cold methanol, collected, and further dried under vacuum. The copolymer structures were then characterized by ^1^HNMR, FTIR, and GPC (Fig. S1–S4, SI).

The thermal properties of NDI based copolymers were evaluated by thermogravimetric analysis (TGA) and differential scanning calorimetry (DSC). We select temperature of 5% weight-loss as the onset point of decomposition (*T*_d_). As shown in Fig. S5, both copolymers show excellent stability with a decomposition temperature of 330 °C for PNDI-2T and 374 °C for PNDI-2TF. No distinct phase transition was observed in the DSC curves of PNDI-2T and PNDI-2TF, revealing the absence of significant degrees of bulk melting crystallinity or phase transition across the measured temperature range (Fig. S6, SI).

### 2.2. Optical and electronic properties

**Fig. 1:**
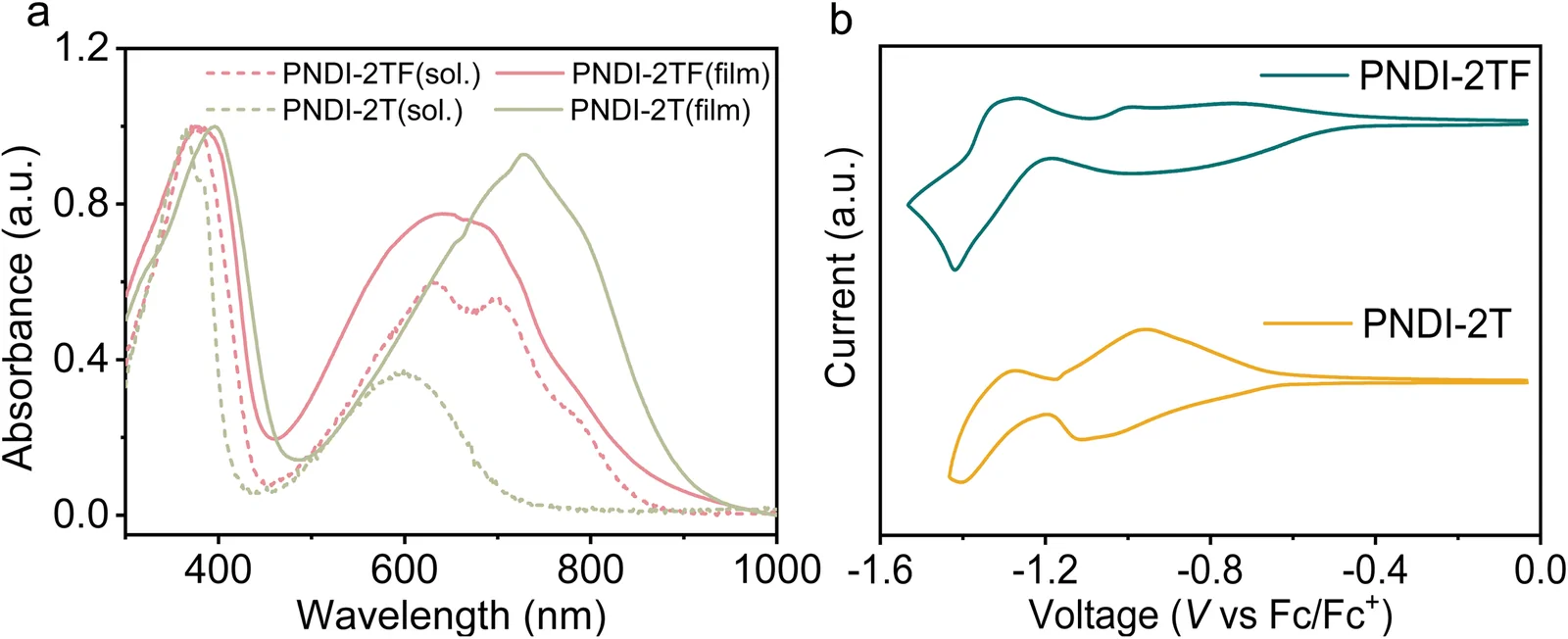
(a) Normalized UV-Vis absorption spectra of polymers PNDI-2T and PNDI-2TF in diluted chloroform solutions (10^−5^ M) and as solid thin films. (b) Cyclic voltammograms of the NDI-based conjugated polymer thin films deposited on glass carbon working electrode immersed in 0.1 M *n*-Bu_4_PF_6_ acetonitrile solution at 100 mVs^−1^.

Fig. 1a presents the ultraviolet-visible-near-infrared (UV-Vis-NIR) absorption spectra of PNDI-2T and PNDI-2TF in dilute chloroform solution and thin film state, and their corresponding optical properties parameters are summarized in Table 1. Both PNDI-2T and PNDI-2TF show the typical two characteristic absorption: a high-energy π–π* transition (300 nm to 450 nm) and a broad, low-energy intramolecular charge-transfer (ICT) transition (450 nm to 900 nm) resulting from the donor–acceptor structure of the polymers. From solution to film, both polymer thin films exhibited red-shifted and broaden absorption spectra as a result of the enhanced polymer interchain π–π stacking and aggregation in the solid state. PNDI-2 TF exhibits an absorption maximum (*λ*_max_) at 646 nm while PNDI-2T shows a bathochromically shifted *λ*_max_ at 729 nm in the thin film state, indicating that the fluorine-substituted on polymer backbone leads to an enlarged band gap. Based on the film absorption onsets, the optical band gaps (*E*_g_^opt.^) are estimated to be 1.39 and 1.44 eV for PNDI-2T and PNDI-2TF, respectively.

**Table 1:** Optical, electrochemical, and energy-level properties of the NDI-based copolymers

Polymers	*λ*_max_^sol.^ (nm)	*λ*_max_^film^ (nm)	*λ*_onset_^film^ (nm)	aa *E*_g_^opt.^ (eV)	*E*_onset_^red.^ (V)	bLUMO (eV)	cHOMO (eV)
PNDI-2T	600	729	890	1.39	−0.64	−4.16	−5.43
PNDI-2TF	629	646	833	1.44	−0.49	−4.31	−5.75

a Optical bandgap calculated using onset of the thin film absorption spectra (*E*_g_^opt.^ = 1240/*λ*_onset_^film^).

b Calculated from CV reduction recorded in a CHCN_3_ solution: *E*_LUMO_ = −(5.10 + *E*_onset_^red.^) eV.

c Calculated from E_LUMO_ and *E*_g_^opt.^: *E*_HOMO_ = *E*_LUMO_ + *E*_g_^opt.^.

In order to gain an insight into the effects of fluorination on the frontier molecular orbital energy levels, we performed cyclic voltammetry (CV) on the polymers in thin film in 0.1 M *n*-Bu_4_NPF_6_ in dry acetonitrile using Ag/AgCl as a pseudeo-reference electrode *versus* Ferrocene/ferrocenium (Fc/Fc^+^). The resulting plots are shown in Fig. 1b and S7, and the corresponding data are summarized in Table 1. The reduction onset was −0.64 V for PNDI-2T and −0.49 V for PNDI-2TF, which are relative to the redox potential of Fc/Fc^+^. The estimated LUMO energy levels are calculated to be −4.16 eV for PNDI-2T and −4.31 eV for PNDI-2TF. Based on the optical band gap (*E*_g_^opt.^) and LUMO levels, we calculated the highest occupied molecular orbital (HOMO) levels by subtracting optical band gap (*E*_g_^opt.^) from LUMO levels to give the value −5.43 eV for PNDI-2T and −5.75 eV for PNDI-2TF. These results reveal that fluorinated polymer backbone pushes down the energy levels, which stems from the inductive effect of the strong electron-withdrawing F atoms on the conjugated polymer backbone, highlighting that backbone engineering is an effective method to modulate the electronic properties of the semiconducting polymers. Both polymers exhibited deep-lying LUMO level (<−4.0 eV), which are beneficial for electron injection and electrochemical doping. The deeper LUMO levels of PNDI-2TF therefore promote a more facile reduction process.

Density functional theory (DFT) calculations were performed to gain insight into the impact of fluorinated polymer backbone on the electronic structure of NDI-based conjugated polymers. One repeat unit of each NDI-based polymer was used to simplify the calculation at the B3LYP/6-31G(d,p) level using Gaussian 16. As shown in Fig. S8, the optimized geometries of both NDI-based monomers exhibit highly twisted structures in gas-phase calculations. The twists backbone mainly occurred between the NDI core and the adjacent thienyl groups. Both the HOMO orbitals and LUMO orbitals are isolated on their donor and acceptor moieties, respectively, in both the NDI based copolymers. The insertion of fluorine atoms into the bithiophene units leads to lower energy levels, a result that is consistent with the CV investigation trend.

### 2.3. OECT device fabrication and characterization

**Fig. 2:**
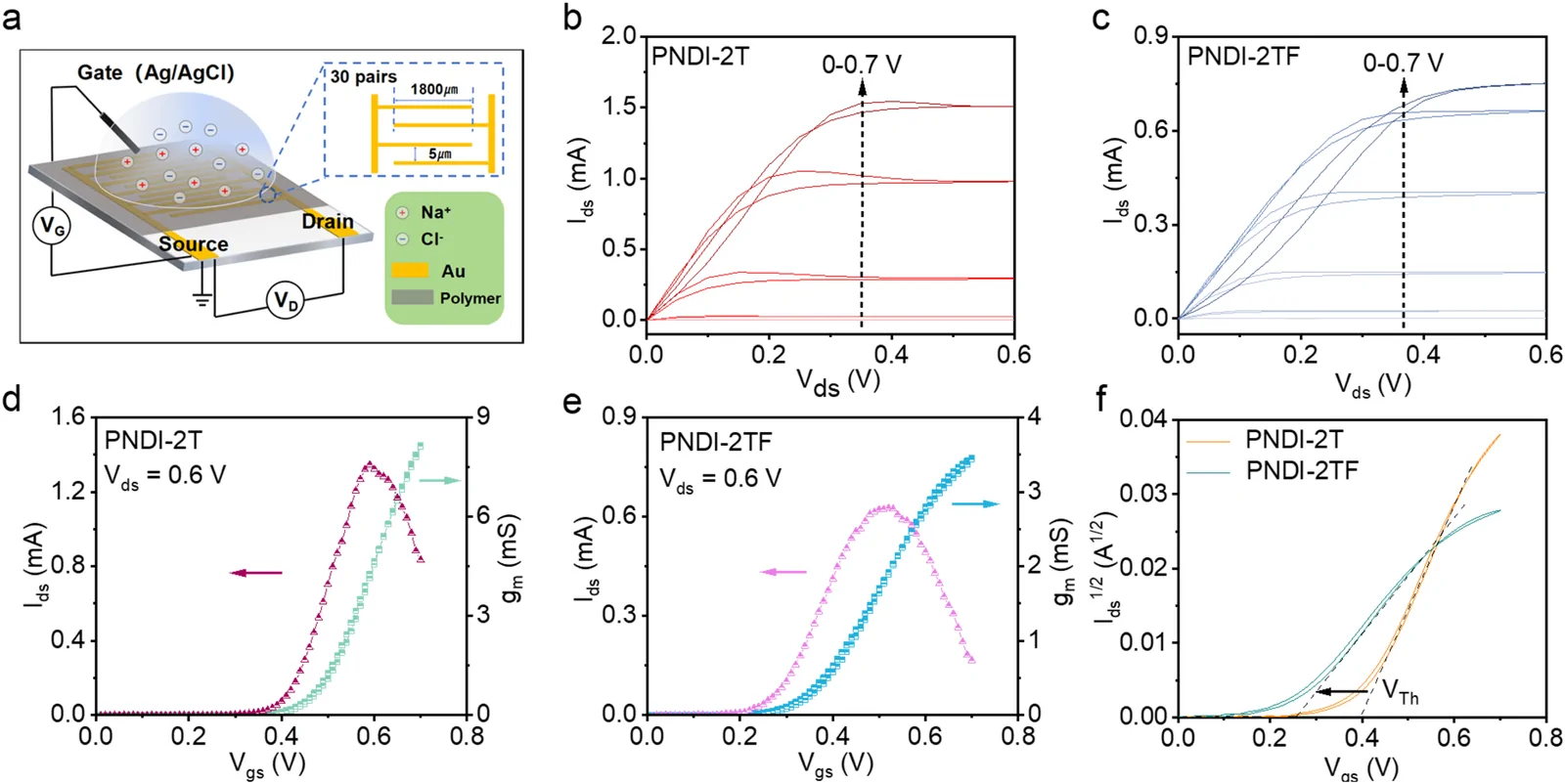
(a) Transfer and (c) output curves of the PNDI-2TF-based OECTs. (b) Transfer and (d) output curves of the PNDI-2T-based OECTs. (e) V_Th_ of NDI-based conjugated polymers. (f) V_g_-dependent transconductance curves of the NDI-based conjugated polymers.

Subsequently, we fabricated OECT devices by employing interdigitated electrodes as source and drain (channel length of 5 µm, widths of 1.8 mm, 30 pairs, MicruX technologies) and using PNDI-2T and PNDI-2TF as the channel active materials to evaluate their OECT performance and investigate the effect of fluorinated donor functionalization on the mixed ionic and electronic conduction properties, as illustrated in Fig. 2 The conjugated polymers were deposited *via* spin-coating from chloroform solutions, without requiring additives or subsequent annealing treatments. We then evaluated the device performance in an aqueous 100 mM NaCl solution using an Ag/AgCl electrode as the gate electrode. The details of devices fabrication and measurement are provided in the SI.

The output and transfer characteristics of both polymers are shown in Fig. 2 and the corresponding OECT parameters are summarized in the Table 2. Both polymer-based OECT devices exhibited typical n-type transport characteristic curves in the accumulation mode upon applying positive gate voltages and demonstrated large on/off ratio of approximately 10^5^. PNDI-2 TF with a donor fluorinated exhibited slightly lower device performance compared to PNDI-2T. Specifically, PNDI-2TF achieved a maximum drain current of 0.78 mA at *V*_G_ = 0.7 V and *V*_D_ = 0.6 V, which is lower than the 1.45 mA measured for PNDI-2T under identical bias conditions (*V*_G_ = 0.7 V, *V*_D_ = 0.6 V). Consequently, the peak transconductance (*g*_m_) values of PNDI-2T and PNDI-2TF are calculated to be 6.88 and 2.77 mS, respectively, yielding unprecedented geometry-normalized transconductance (*g*_m,norm_) of 0.034 and 0.019 S cm^−1^, respectively, calculated as *g*_m,norm_ = *gmL*/*Wd*. The µC* values of PNDI-2T and PNDI-2TF were calculated as 0.197 F cm^−1^ V^−1^ and 0.074 F cm^−1^ V^−1^, respectively, according to the eqn (1). The threshold voltage (*V*_Th_) of OECT is another important parameter besides the transconductance, which is predominantly dominated by the responsiveness of the film to ion permeation and the energy levels of active channel materials. As shown in Fig. 2f, the PNDI-2T based OECT displayed a higher n-type threshold voltage *V*_Th_ of 0.386 V, while the donor with fluorine functionalization in PNDI-2TF downshifted the LUMO level, resulting in reducing *V*_Th_ to 0.232 V. The lower threshold voltage is beneficial for OECT based complementary logic circuits and chemical sensors, because it critically impacts the device's energy efficiency and noise tolerance during operation.

**Table 2:** Aqueous electrolyte gated OECT characteristics of the NDI based copolymers

Polymer	*d* (nm)	*g*_m_ (mS)	a *g*_m,norm_ (S cm^−1^)	*I*_on/off_	b *V*_Th_ (V)	c µC* (F cm^−1^ V^−1^ s^−1^)	d C* (F cm^−3^)	e *µ*_e_ OECT (cm^2^ V^−1^ s^−1^)
PNDI-2T	95	6.88 ± 0.3	0.034 ± 0.001	1.4 × 10^5^	0.386 ± 0.004	0.182	93.8	2.1 × 10^−3^
PNDI-2TF	67	2.77 ± 0.08	0.019 ± 0.001	0.77 × 10^5^	0.232 ± 0.004	0.076	48.4	1.5 × 10^−3^

a The maximum transconductance values were normalized by the channel geometry.

b Values obtained by extrapolating the corresponding *I*_ds_^1/2^–*V*_g_ plots.

c Values obtained from the equation: 
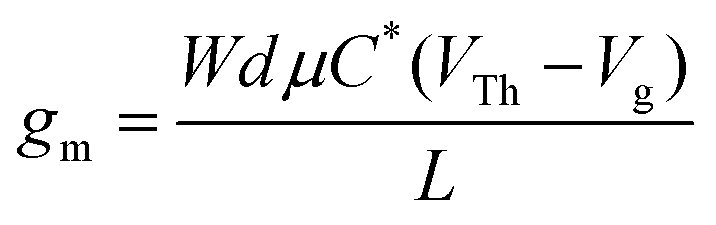
. Dimensions of the interdigitated electrodes (IDA-Au-6, *L* = 5 µm, *w* = 1.8 mm, pairs = 30), *Wd*/*L* = 0.108 cm.

d Values deduced from electrochemical impedance spectroscopy curves fit to an *R*_s_ (*R*_p_//*C*) circuit.

e Values calculated from the µC* and the volumetric capacitance C*.

### 2.4. Spectroelectrochemical, wettability and molecular parking property characterization

To gain insight into the performance differences of these two polymers in OECTs, we investigated their electrochemical properties (*e.g.*, redox potentials, volumetric capacitance, UV-Vis-NIR spectroelectrochemistry) in 100 mM NaCl solution, molecular packing, and wettability to understand the influence of fluorine substitution in the donor of D-A conjugated polymers.

**Fig. 3:**
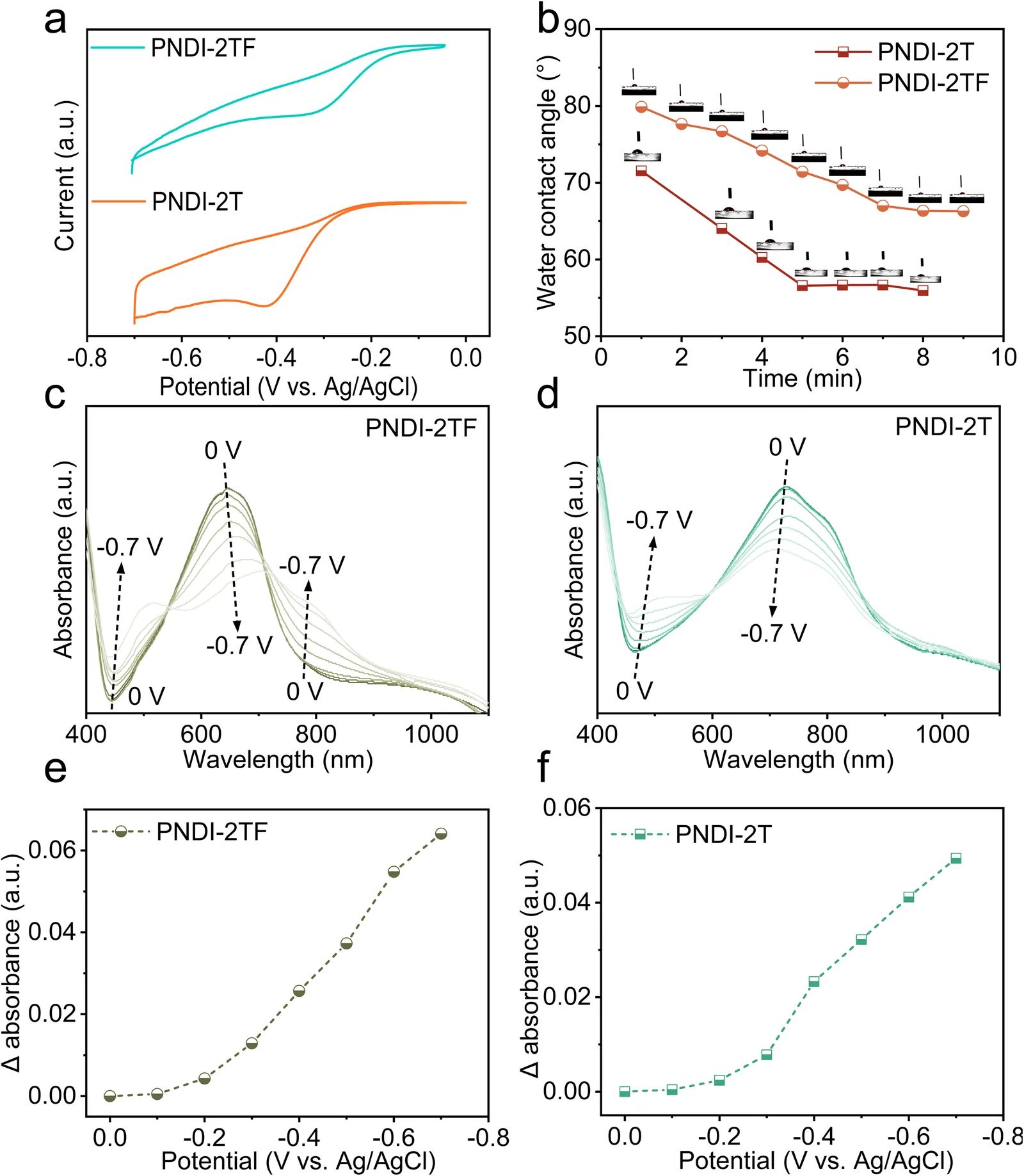
(a) Cyclic voltammograms of the NDI-based conjugated polymer thin films deposited on ITO electrode immersed in in 0.1 M NaCl aqueous solution at 50 mV s^−1^. (b) Water contact angle of NDI-based conjugated polymer thin film over the time. (c and d) UV-Vis-NIR spectroelectrochemistry of PNDI-2T and PNDI-2TF thin films in 0.1 M NaCl aqueous solution at different bias against Ag/AgCl reference. (e and f) Absolute change of PNDI-2T and PNDI-2TF ICT absorption under applied bias.

We first carried out a combination of cyclic voltammetry (CV), UV-Vis-NIR spectroelectrochemistry and electrochemical impedance spectroscopy (EIS) measurements in 100 mM NaCl solution to gain insight into the electrochemical property behavior of both conjugated polymers. As shown in Fig. 3a, both conjugated polymers exhibited reduction behavior with onset potentials at −0.28 V and −0.11 V (*vs.* Ag/AgCl) for PNDI-2T and PNDI-2TF in aqueous solution, respectively, which shows a trend consistent with that observed in organic solution (*e.g.*, acetonitrile). This result demonstrates that the cations can more easily penetrate the PNDI-2TF film than into the PNDI-2T because the electron-withdrawing F atoms downshift the LUMO level. Beyond the energy level factor, the polymer film wettability also plays an important role in ion penetration and transport. The contact angles of both thin films were tested over time, and the results are shown in Fig. 3b. It was observed that the contact angle of PNDI-2TF film remained consistently higher than that of PNDI-2T film throughout the wetting period. This difference clearly indicates that PNDI-2TF exhibits enhanced hydrophobicity, primarily attributed to the hydrophobic nature of fluorine atoms. This observation is correlated with the recently published report.^40^ Notably, despite its elevated hydrophobicity, which typically impedes ion uptake. PNDI-2TF still demonstrated efficient ion uptake and transport during electrochemical doping in an aqueous environment. To gain insight into the this phenomenon, we carried out the electrochemical impedance spectroscopy (EIS) measurements to experimentally derived from the volumetric capacitance (C*). As seen in Fig. S9–11, PNDI-2TF exhibits a lower C* value (48.4 F cm^−3^) compared to PNDI-2T (93.8 F cm^−3^). This lower volumetric capacitance highly result from the increased hydrophobicity. Collectively, these findings demonstrate that the incorporation of an oligo(ethylene glycol) (OEG) side chain segment into the polymer is sufficient to facilitate ion uptake, even in the presence of hydrophobic moieties. In addition, based on volumetric capacitance (C*) and µC* values, the electron mobility (*µ*) was determined to be 2.1 × 10^−3^ cm^2^ V^−1^ s^−1^ for PNDI-2T and 1.5 × 10^−3^ cm^2^ V^−1^ s^−1^ for PNDI-2TF, respectively. Therefore, the reduced electron mobility and lowered volumetric capacitance of PNDI-2TF ultimately compromise the OECT device performance, manifesting as lower transconductance.

To investigate the electrochemical doping kinetics of both polymer films in a 100 mM NaCl aqueous solution, spectroelectrochemical measurements were performed to record the absorbance spectra of the films under a successive step potential bias. As shown in Fig. 3c and d, both polymer films underwent electrochemical reduction as the bias was swept from 0 to −0.7 V, which was evidenced by a gradual decrease in the intensity of the original transition bands and a gradual increase in the intensity of the polaron bands. Specifically, PNDI-2T gradually lost its original absorption peak at 748 nm and exhibited an increased polaron/bipolaron absorption at 500 nm and 910 nm. PNDI-2 TF gradually lost its original absorption peak at 625 nm and exhibited an increased polaron/bipolaron absorption at 500 nm and 800 nm. Such observations verify the n-type characteristic of both polymers. Generally, the reduction in intensity of original peak is proportional to the concentration of polaron carriers generated through electrochemical doping. As shown in Fig. 3e and f, we plotted the relative variations in the intensity original absorption peak of films over the applied bias. The original absorption band began to decrease at −0.2 V for PNDI-2TF, which is relatively lower than that of PNDI-2TF (−0.3 V), indicating an earlier doping onset, due to the decreased LUMO energy level of PNDI-2TF. Furthermore, the PNDI-2TF film exhibits a large loss in intensity compared to PNDI-2T at same bias voltage, revealing high doping level of PNDI-2TF than that of PNDI-2T. The high doping of PNDI-2TF is not directly translated to higher OECT device performance; we suspect that it is highly possible that this originates from reduced charge mobility and enhanced hydrophobicity, which limit ion penetration.

**Fig. 4:**
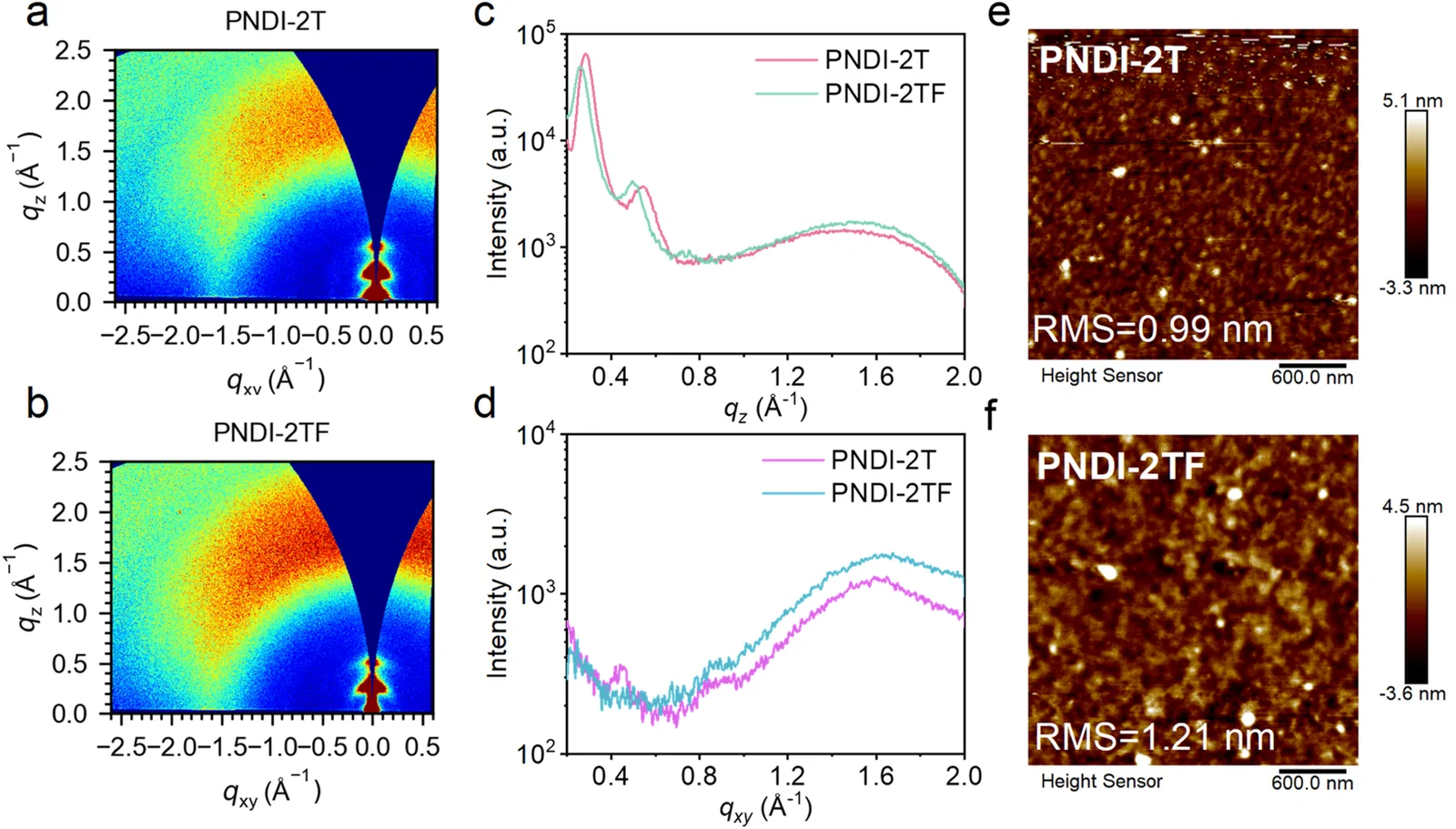
(a and b) 2D GIAWXS images of PNDI-2T and PNDI-2TF; (c and d) line-cut profiles of PNDI-2T and PNDI-2TF thin film by integration along the in-plane (*q*_*r*_) and out-of-plane (*q*_*z*_) direction; (e and f) AFM images of PNDI-2T and PNDI-2TF films.

**Fig. 5:**
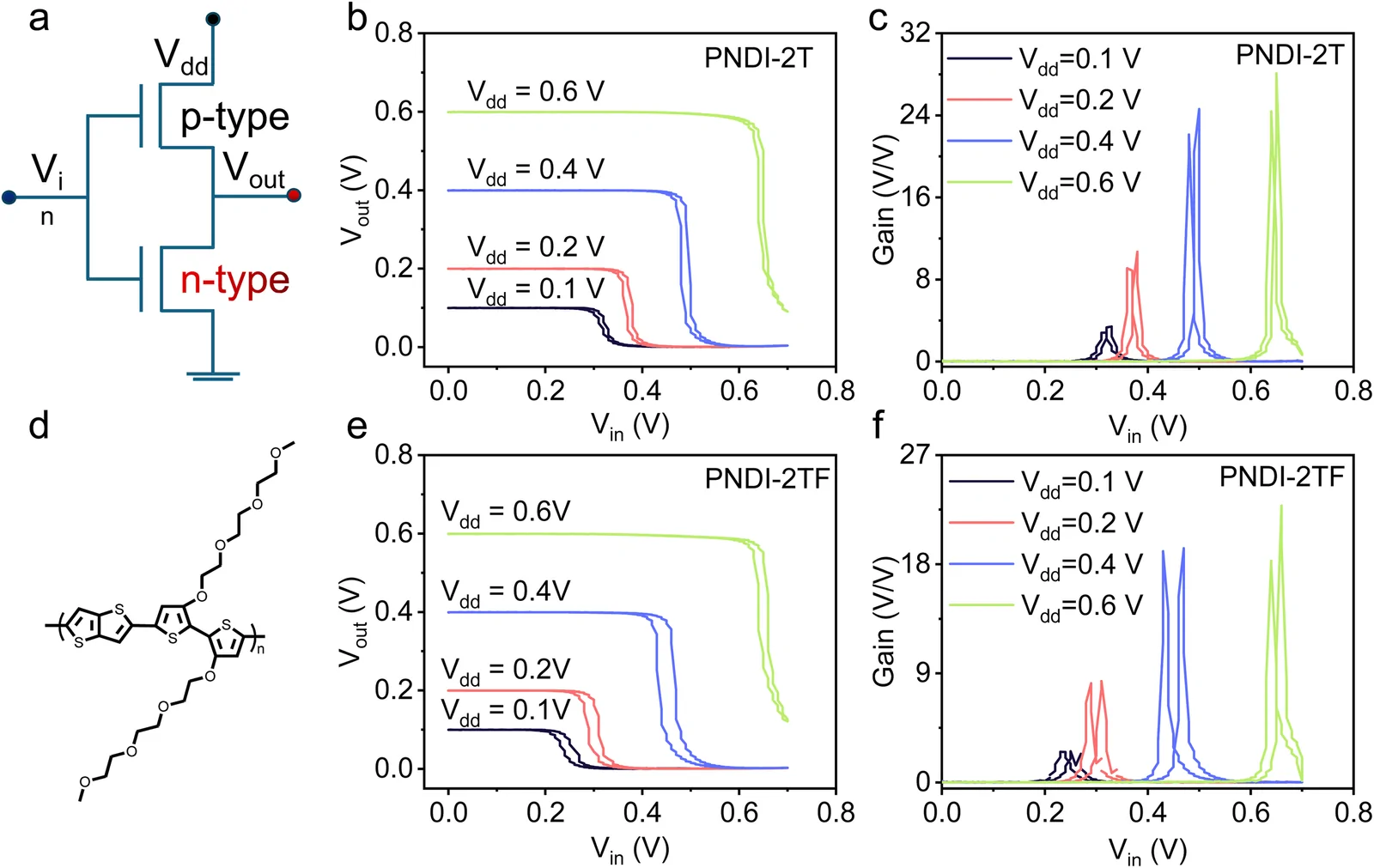
(a) A schematic representation of the complementary inverter using Pg2T-TT as p-type channel and NDI-based conjugated polymers n-type channel, 100 mM NaCl as electrolyte. (b) Chemical structure of the polymer Pg2T-TT. (c) Input–output characteristics of a typical complementary-like inverter using PNDI-2T as n-type channel and (d) corresponding voltage gains (|∂*V*_out_/∂*V*_in_|) of the inverters. (e) Input–output characteristics of a typical complementary-like inverter using PNDI-2TF as n-type channel and (f) corresponding voltage gains (|∂*V*_out_/∂*V*_in_|) of the inverters.

We subsequently performed the grazing-incidence wide-angle X-ray scattering (GIWAXS) and atomic force microscope (AFM) measurement to further investigate the effects of fluorine substitution on polymer film crystallinity, molecular packing and film morphology. The two-dimensional (2D) patterns and corresponding line-cut profiles of PNDI-2T and PNDI-2TF as presented in Fig. 4a–c and 5d–f. Both polymers exhibit edge-on dominated orientation, as evidenced by the strong and highly ordered molecular stacking indicated by lamellar diffraction peaks of (100) and (200) in the out-of-plane (OOP) direction, along with a (010) diffraction peak in the in-plane (IP) direction. The PNDI-2T film exhibited lamellar diffractions (100) at *q*_*z*_ = 0.286 Å^−1^ and (200) peak at *q*_*z*_ = 0.545 Å^−1^ in the OOP direction, along with a π–π stacking peak at *q*_*xy*_ = 1.609 Å^−1^ in the IP direction. While PNDI-2TF film showed lamellar diffractions (100) at *q*_*z*_ = 0.261 Å^−1^ and (200) peak at *q*_*z*_ = 0.498 Å^−1^ in the OOP direction, along with a π–π stacking peak at *q*_*xy*_ = 1.623 Å^−1^ in the IP direction. The lamellar distance of PNDI-2T and PNDI-2TF were 21.95 and 24.06 nm, respectively, as calculated from the (100) peaks in OOP direction. The π–π stacking distances of neat films of PNDI-2T and PNDI-2TF were 3.90 and 3.87 nm, respectively, as calculated from the (010) peaks in IP direction. The crystal coherence lengths (CCLs) of π–π stacking and lamellar stacking are 8.9 and 85.1 nm for neat films of PNDI-2T, and 7.3 and 72.6 nm for neat films of PNDI-2TF, respectively. The reduced CCLs and higher *g* were obtained in both the π–π stacking and lamellar stacking structures for PNDI-2TF, indicating more disordered molecular packing of PNDI-2TF relative to PNDI-2T, which could be understood from PNDI-2TF slightly twisted molecular backbone manifested in DFT calculations. Thus, the unfavorable molecular packing and reduced crystallinity of the PNDI-2TF film are the main contributing factors for poor charge carrier transport properties, which correlate with its low OECT devices performance. In the AFM height images, both spin-coated polymer films exhibit smooth surfaces with low root-mean-square (RMS) roughness values of 0.99 nm for PNDI-2T and 1.21 nm for PNDI-2TF (Fig. 4e and f), respectively, and no distinct surface nanostructures are observed. Such smooth surface morphologies indicate superior film quality, which is conducive to realizing high device performance.

To further elucidate the effects of fluorine substitution on polymer film charge transport in pristine conjugated polymers, we fabricated bottom-gate/bottom-contact organic field-effect transistors (OFETs) with a device structure of doped silicon/neat polymer/Al (Fig. S12, SI). The corresponding output and transfer characteristics of the OFETs are displayed in Fig. S13. PNDI-2T exhibited n-type unipolar charge transport and the electron mobilities of PNDI-2T was 1.25 × 10^−2^ cm^2^ V^−1^ s^−1^. However, it was not possible to measure this parameter for PNDI-2TF under same experimental conditions. This suppressed charge transport behavior induced by fluorination is not unique in our PNDI-2TF polymer and has been widely documented in analogous NDI-based conjugated polymers due to the enhanced increased structural disorder severely disrupts intermolecular π–π stacking and charge hopping pathways. The lower OFET/OECT charge mobility of PNDI-2TF than that of PNDI-2T can be explained by the poor polymer chain packing of the former in the thin films, which was consistent with the GIWAXS data. The discrepancies in charge transport observed between OFET and OECT, as they differ fundamentally in their charge accumulation mechanisms, carrier density level, transport-limiting factors, and device operating regimes.

### 2.5. Complementary inverters

OECT-based complementary inverters have garnered increasing attention in recent years, owing to their promising potential for applications in logic circuits and the amplification of electrophysiological signals in bioelectronics and flexible electronics.^48,49^ The schematic of the OECT-based complementary accumulation-mode inverter is shown in Fig. 4a, in which polythiophene functionalized with oligoethylene glycol side chains (P(g2T-TT)) and NDI-based polymers (PNDI-2T or PNDI-2TF) were used as the p-type and n-type semiconducting polymers, respectively. The voltage output and gain characteristics of these complementary inverters are presented in Fig. 4(c–f). When the supply voltage (*V*_DD_) is set to 0.6 V and the input voltage (*V*_in_) is swept from 0 to 0.7 V, the PNDI-2T-based inverter achieves a maximum gain of 28.3 V/V at 0.7 V (Fig. 4c and d), while the PNDI-2TF-based inverter achieves a maximum gain of 22.9 V/V at 0.7 V (Fig. 4e and f). The inferior performance of the PNDI-2TF-based inverter is largely attributed to the lower OECT performance of PNDI-2TF itself, indicating that obtaining high performance n-type conjugated polymer to match that of p-type conjugated polymer is the key to achieving high-performance complementary OECT inverters.

### 2.6. N-type OECTs for glucose sensors

**Fig. 6:**
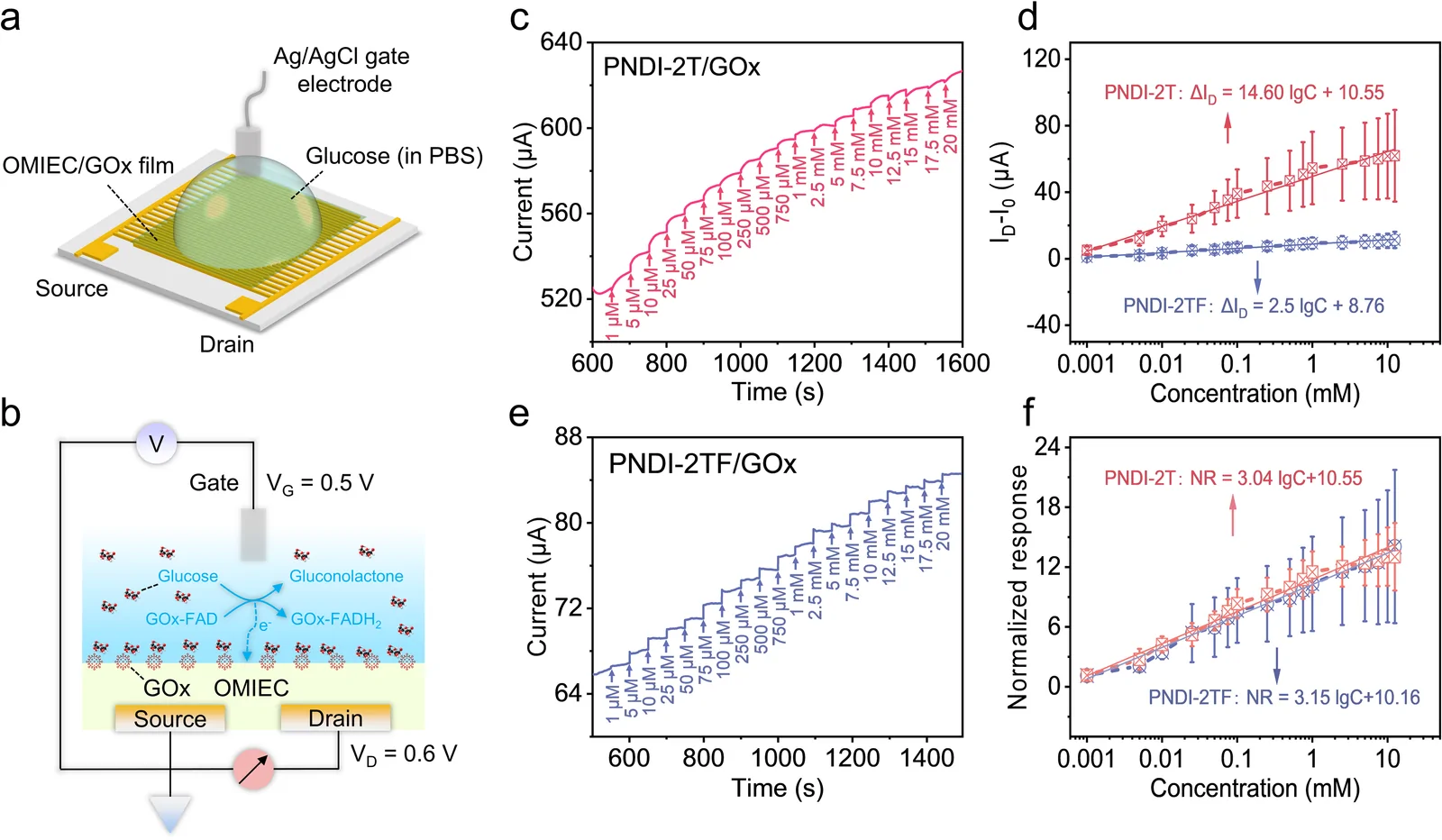
(a) Schematic diagram of the process as a glucose sensor by OECT based on NDI-based polymer. (b) The working mechanism of the glucose sensor. Real-time glucose response of (c) PNDI-2T/GOx- and (e) PNDI-2TF/GOx-modified OECTs gated by Ag/AgCl, recorded upon sequential addition of glucose solutions with increasing concentrations. (d) Corresponding glucose current responses and (f) normalized glucose responses of PNDI-2T/GOx and PNDI-2TF/GOx OECTs gated by Ag/AgCl.

To investigate the effect of fluorine (F) substitution on conjugated polymers in organic electrochemical transistors (OECTs) for glucose biosensors, glucose oxidase (GOx) was used to modify the gate electrode for sensing (see Supporting Information and Fig. 6a). The operating mechanism of the glucose sensor is schematically illustrated in Fig. 6b. Briefly, we employed the n-type polymer (PNDI-2T or PNDI-2TF) films integrated with a GOx-adsorbed, modified channel electrode, in which the n-type polymer film accepts electrons from the enzymatic reaction and directly transfers them to the channel electrode, leading to increased drain currents, which serves as the electrical signal response in the corresponding OECT-based sensors for glucose sensing. The drain current–time responses recorded at a drain voltage of 0.6 V exhibited a pronounced concentration-dependent increase upon successive additions of glucose (1 µM to 20 mM) in the presence of glucose oxidase (GOx) (Fig. 6c and e). A strong linear relationship between drain current and glucose concentration was observed (Fig. 6d and f), with coefficients of determination (*R*^2^) of 0.9781 for PNDI-2T/GOx and 0.9907 for PNDI-2TF/GOx. With regard to glucose sensing performance, both PNDI-2T/GOx and PNDI-2TF/GOx sensors exhibited an ultra-wide linear detection range from 1 µM to 20 mM. However, in terms of absolute current response, PNDI-2T/GOx showed a substantially higher sensitivity of 14.6 µA dec^−1^, whereas PNDI-2TF/GOx exhibited a lower sensitivity of 2.5 µA dec^−1^, indicating that the fluorinated polymer-based sensor is less sensitive in absolute terms. To further compare the relative response behavior of the two sensors, the glucose responses were normalized to their initial drain currents. After normalization to the initial drain current, the two sensors displayed nearly overlapping linear fitting curves, with normalized sensitivities of 3.04% dec^−1^ and 3.15% dec^−1^ for PNDI-2T/GOx and PNDI-2TF/GOx, respectively, indicating comparable normalized sensing performance. Although these results demonstrate the feasibility of PNDI-2T- and PNDI-2TF-based OECTs for enzymatic glucose sensing, the current evaluation should be regarded as a proof-of-concept demonstration. One important advantage of this platform is that the EG-containing side chains of the n-type polymers can provide a favorable interface for GOx adsorption, allowing enzymatic glucose sensing to be achieved through a simple physical adsorption process without additional surface modification or complex enzyme immobilization chemistry. This simplified fabrication strategy may be beneficial for the development of polymer-based enzymatic OECT biosensors. Nevertheless, for practical biosensor applications, several additional performance metrics require systematic investigation in future studies, including selectivity against common interfering species, long-term operational and storage stability, device-to-device reproducibility, response time, and sensing performance in complex biological environments. In addition, tests in artificial or real biological fluids, together with repeated measurements over extended periods, will be important for validating the robustness and practical applicability of these OECT-based glucose sensors.

Subsequently, the surface properties of the PNDI-2T and PNDI-2TF films were further examined to understand their possible influence on enzyme adsorption and sensing performance. As shown in Fig. S18, the PNDI-2TF film exhibited slightly reduced wettability and a more negative surface zeta potential compared with PNDI-2T. Previous studies have shown that the surface wettability and charge of n-type polymer films can strongly influence GOx adsorption behavior, including the adsorbed amount, conformation, and possible orientation of the enzyme. In particular, negatively charged polymer surfaces have been suggested to favor a back-lying GOx orientation, which may facilitate the accessibility of the active site and its interaction with the electrode.^20,50^ Inspired by these reports, the more negative surface potential of PNDI-2TF may suggest a more favorable enzyme–polymer interfacial interaction. However, because the enzyme orientation was not directly characterized in this study, the proposed orientation shown in Fig. S19 should be regarded only as a possible interfacial configuration rather than direct structural evidence. Therefore, although fluorine substitution decreases the electron mobility and volumetric capacitance of the conjugated polymer, resulting in lower absolute current sensitivity for the PNDI-2TF/GOx sensor, it may still modulate the biochemical interface through changes in surface wettability and surface charge. These results suggest that fluorination affects both the intrinsic electronic properties of the polymer and the enzyme–polymer interfacial characteristics. This distinction highlights the importance of balancing semiconductor charge-transport properties with surface chemistry when designing OECT-based enzymatic biosensors.

## 3 Conclusion

In summary, we have developed two n-type D-A conjugated polymers (PNDI-2T and PNDI-2TF) based on a naphthalenediimide (NDI)-bithiophene (2T) backbone, both polymers featuring amphipathic side chains and with or without fluorine (F) substitution on the bithiophene unit. Compared to nonfluorinated PNDI-2T, although fluorinated PNDI-2TF exhibits increased hydrophobicity, primarily attributed to the hydrophobic nature of fluorine (F) atoms, fluorinated PNDI-2TF yields a lower-lying LUMO level, due to the strong electron-withdrawing nature of fluorine (F) atoms, which is beneficial for reducing the ion penetration and transporting barrier in OECTs. Consequently, PNDI-2TF-based devices showed a significantly reduced *V*_th_ to 0.25 V compared to PNDI-2T-based ones (0.40 V). *Via* a combination of optical spectroscopy, atomic force microscopy, and 2D grazing incidence wide-angle X-ray scattering characterizations, we unraveled that the lower PNDI-2TF-based OECT performance results from the reduced electron mobility. In addition, we fabricated complementary inverters by pairing the p-type polymer (Pg2T-TT) with the n-type polymers (PNDI-2T or PNDI-2TF), achieving maximum voltage gains of 28.3 and 22.9 V/V for PNDI-2T and PNDI-2TF, respectively, at a supply voltage of 0.7 V. Furthermore, the glucose sensors, which utilizes n-type conjugated polymer (PDNI-2T or PDNI-2TF) as the active layer, both exhibited comparable and an ultra-wide linear detection range from 1 µM to 20 mM for glucose sensing with a good linear response. Overall, this work not only elucidates the influence of fluorination on NDI-based polymers, an insight crucial for validating the fluorine substitution strategy in developing high-performance n-type organic mixed ionic-electronic conductors, but also demonstrates the promising prospects of NDI-based polymers in bioelectronic applications.

## Author contributions

The manuscript was written through contributions of all authors. All authors have given approval to the final version of the manuscript. Xinnian Jiang (data curation: equal; formal analysis: equal; investigation: equal; methodology: equal; writing – original draft: equal), Xiandi Yang (data curation: equal; formal analysis: equal; investigation: equal; methodology: equal; writing – original draft: equal), Jiazheng Li (data curation: equal; formal analysis: equal; investigation: equal; methodology: equal; writing – original draft: equal), Wenhao Zuo (formal analysis: supporting; investigation: supporting; visualization: supporting), Zhi Li (data curation: supporting; visualization: supporting), Man Wang (data curation: supporting; visualization: supporting), Qiaogan Liao (data curation: supporting; visualization: supporting), Junyu Li (data curation: supporting; visualization: supporting), Tiedong Cheng (conceptualization: supporting; project administration: supporting; resources: equal; supervision: supporting), Ping Zhang (conceptualization: equal; funding acquisition: equal; project administration: equal; resources: equal; supervision: equal; writing – review and editing: equal), Yanxi Zhang (conceptualization: equal; funding acquisition: equal; project administration: equal; resources: equal; supervision: equal; writing – review and editing: equal), Gang Ye (conceptualization: equal; project administration: equal; resources: equal; supervision: equal; writing – review and editing: equal).

## Conflicts of interest

The authors declare no competing financial interest.

## Supplementary Material

RA-016-D6RA03225G-s001

## Data Availability

The data supporting this article have been included as part of the supplementary information (SI). Supplementary information: detailed description of the synthesis and characterization of the materials; polymer thin film characterization; OECT devices fabrication and characterization; organic field-effect transistor (OFET) fabrication and characterization; glucose bio-sensors fabrication and characterization; supporting Fig. S1–S19. See DOI: https://doi.org/10.1039/d6ra03225g.
