# Supplementary material for: Influence of fluorine substitution in naphthalenediimide–bithiophene (NDI–2T)-based n-type conjugated polymers for organic electrochemical transistors and glucose biosensors
Source: RSC Adv. 2026 Jul 29;16(39):43778–90. doi: 10.1039/d6ra03225g (PMC13417991; doi:10.1039/d6ra03225g)
Supplement: Supplementary file 1 [file RA-016-D6RA03225G-s001.pdf]

Supplementary Information (SI) for RSC Advances.
This journal is © The Royal Society of Chemistry 2026

Supporting information

Influence of Fluorine-Substituted in Naphthalenediimide (NDI)-bithiophene (2T)-based n-Type Polymer Semiconductors for Organic Electrochemical Transistors and Glucose Bio-Sensors

Xinnian Jiang‡[b], Xiandi Yang‡[a], Jiazheng Li‡[b], Wenhao Zuo[c], Zhi Li[b], Man Wang[b], Qiaogan Liao[d], Junyu Li[e], Tiedong Cheng[a,f], Ping Zhang*[a,f], Yanxi Zhang*[b], Gang Ye*[c]

Xiandi Yang, Tiedong Cheng, Ping Zhang,

[a] School of Electrical Engineering and Automation, Jiangxi University of Science and Technology, Ganzhou, Jiangxi, 341000, China

Email: p.zhang@jxust.edu.cn

Xinnian Jiang, Jiazheng Li, Zhi Li, Man Wang, Yanxi Zhang

[b] Institute of Flexible Electronics (IFE, Future Technologies), Xiamen University, Xiamen 361005, China.

E-mail: ifeyxzhang@xmu.edu.cn

Wenhao Zuo, Gang Ye

[c] Ministry of Education Key Laboratory for the Green Preparation and Application of Functional Materials, Hubei Key Laboratory of Polymer Materials, School of Materials Science and Engineering, Hubei University, Youyi Road 368, Wuhan 430062, P. R. China

E-mail: g.ye0612@hubu.edu.cn

Qiaogan Liao

[d] School of Materials Science and Engineering, Guilin University of Electronic Technology, Guilin, Guangxi, 541004, China

Junyu Li

[e] Sinopec Shanghai Research Institute of Petrochemical Technology, Shanghai 201028, China.

Tiedong Cheng, Ping Zhang

[f] Jiangxi Provincial Key Equipment Industry Technology Engineering Center for Microgrids, Ganzhou, Jiangxi, 341000, China

‡ X. J., X. Y. and J. L. contribute to this work equally.

1. Materials synthesis and characterization

Reagents

All reagents and solvents were commercial and were used as received. 4,9-dibromo-2,7-di(2,5,8,11-tetraoxanonadecan-19-yl)benzo[lmn][3,8]phenanthroline-1,3,6,8(2H,7H)-tetraone (**NDIC8TEG**) was synthesized according to literature procedures[1]. 5,5'-bis(trimethylstannyl)-2,2'-bithiophene (**2T**) (3,3'-difluoro-[2,2'-bithiophene]-5,5'-diyl)bis(trimethylstannane) (**2TF**) were purchased from Suna Tech Inc.

Glucose oxidase (GOx, ≥10,000 U/g solid) was purchased from Sigma-Aldrich. β-D-Glucose (≥ 85%, Rhawn) was used as the target analyte in this study. Phosphate-buffered saline (PBS, 10×, Acmec) was employed to prepare glucose solutions of various concentrations. Indium tin oxide (ITO) glass substrates used during the experiments were obtained from South China Xiangcheng Technology Co., Ltd. Quartz glass slides (1 cm × 2 cm × 1 mm) for zeta-potential measurements were supplied by Spectrum Analysis Optical Components Co., Ltd. Gold interdigitated electrodes were provided by Micrux Technologies. Prior to use, the ITO glass, quartz slides, and IDEs were treated using a UV-ozone cleaner to remove surface contaminants. A solid-state Ag/AgCl sintered electrode (diameter: 2 mm; height: 4 mm), customized for use as the gate electrode, was manufactured by Wuhan Brainlink Technologies Co., Ltd. A saturated Ag/AgCl reference electrode (R218) and a platinum plate electrode (PT211, 1 cm × 1 cm × 0.2 mm) were obtained from Shanghai Yueci Electronic Technology Co., Ltd.

Characterization

^{1}HNMR were performed on a Varian Unity Plus (400 MHz) instrument at 25 °C, using tetramethylsilane (TMS) as an internal standard. NMR shifts are reported in ppm, relative to the residual protonated solvent signals of $CDCl_3$ (δ= 7.26 ppm) or at the carbon absorption in $CDCl_3$ (δ = 77.23 ppm). GPC measurements were done on a Agilent GPC-PL220 room temperature GPC/SEC system at 30 °C vs polystyrene standards using hexafluoroisopropanol (HFIP) as eluent. IR measurements were performed on a Nicolet iS50 FT-IR spectrometer. FT-IR spectra were recorded on a Nicolet Nexus FT-IR fitted with a Thermo Scientific Smart iTR sampler. Thermal properties of the polymers were determined on a TA Instruments DSC Q20 and a TGA Q50. DSC measurements were executed with two heating-cooling cycles with a scan rate of 10 °C min^{-1}, and from each scan, the second heating cycle was selected. TGA measurements were done from 20 to 800 °C with a heating rate of 10 °C min^{-1}. Cyclic voltammetry (CV) measurements in organic solution was carried out with a CHI760 Evoltammetric potentiostat in a three-electrode configuration where the working electrode was glass carbon electrode, the counter electrode was a platinum wire, and the pseudo-reference was an Ag/AgCl wire that was calibrated against ferrocene (Fc/Fc^+). Cyclic voltammograms for NDI-Based polymers film deposited on the glass carbon working electrode in CH_3CN solution containing Bu_4NPF_6 (0.1 mol L^{-1}) electrolyte at a scanning rate of 100 mV s^{-1}.

General synthetic procedures for the NDI based polymers

To a dry three-neck flask, NDI-based monomer (0.1 mmol) and thiophene based monomer (0.1 mmol) were added under argon followed by tris(dibenzylideneacetone) dipalladium [$Pd_2(dba)_3$] (8 mg) and tri(o-tolyl)phosphine [$P(o\text{-}tolyl)_3$] (12 mg). The flask and its contents were subjected to 3 pump/purge cycles with N_2 followed by addition of anhydrous, degassed chlorobenzene (5

mL) via syringe. The reaction mixture was stirred at 110 ℃ for 24h. After cooling to room temperature, the deeply colored reaction mixture was dropped into 100 ml vigorously stirred methanol (containing 5 ml 12 M hydrochloride acid). After stirring for 4 h, the precipitated solid was collected by filtration. The solid polymers were re-dissolved in chloroform and reprecipitated into methanol. After filtration, the polymers were subjected to sequential Soxhlet extraction. The sequential solvents were methanol, hexane and chloroform. Impurities and low-molecular-weight fraction were removed by methanol. Finally, the polymer solution in hexane or chloroform was concentrated to give the polymer as a dark solid.

PNDI-2T Synthesis according to the general polymerization procedure: monomer **NDIC8TEG** (97 mg, 0.1 mmol), monomer **2T** (50 mg, 0.1 mmol), dry chlorobenzene (5 mL). The polymer was obtained as a dark solid (55 mg, 55 %). ^{1}HNMR (400 MHz, $CDCl_3$): δ 8.91-8.48 (m, 2H), 7.58-6.92 (m, 4H) 4.46-3.25 (m, 38H), 1.98-0.97 (m, 32H). IR (cm^{-1}): 689, 718, 765, 790, 930, 1023, 1096, 1196, 1257, 1296, 1309, 1348, 1378, 1438, 1569, 1664, 1701, 2854, 2924.

PNDI-2TF Synthesis according to the general polymerization procedure: monomer **NDIC8TEG** (97 mg, 0.1 mmol), monomer **2TF** (53 mg, 0.1 mmol), dry chlorobenzene (5 mL). The polymer was obtained as a dark solid (68 mg, 65 %). ^{1}HNMR (400 MHz, $CDCl_3$): δ 8.85-8.68 (m, 2H), 7.72-7.01 (m, 2H) 4.39-3.24 (m, 38H), 1.85-1.12 (m, 32H). IR (cm^{-1}): 654, 708, 725, 765, 793, 843, 929, 996, 1102, 1195, 1243, 1313, 1367, 1403, 1439, 1535, 1571, 1662, 1704, 2855, 2924.

2. ^{1}HNMR spectra of NDI-based copolymers

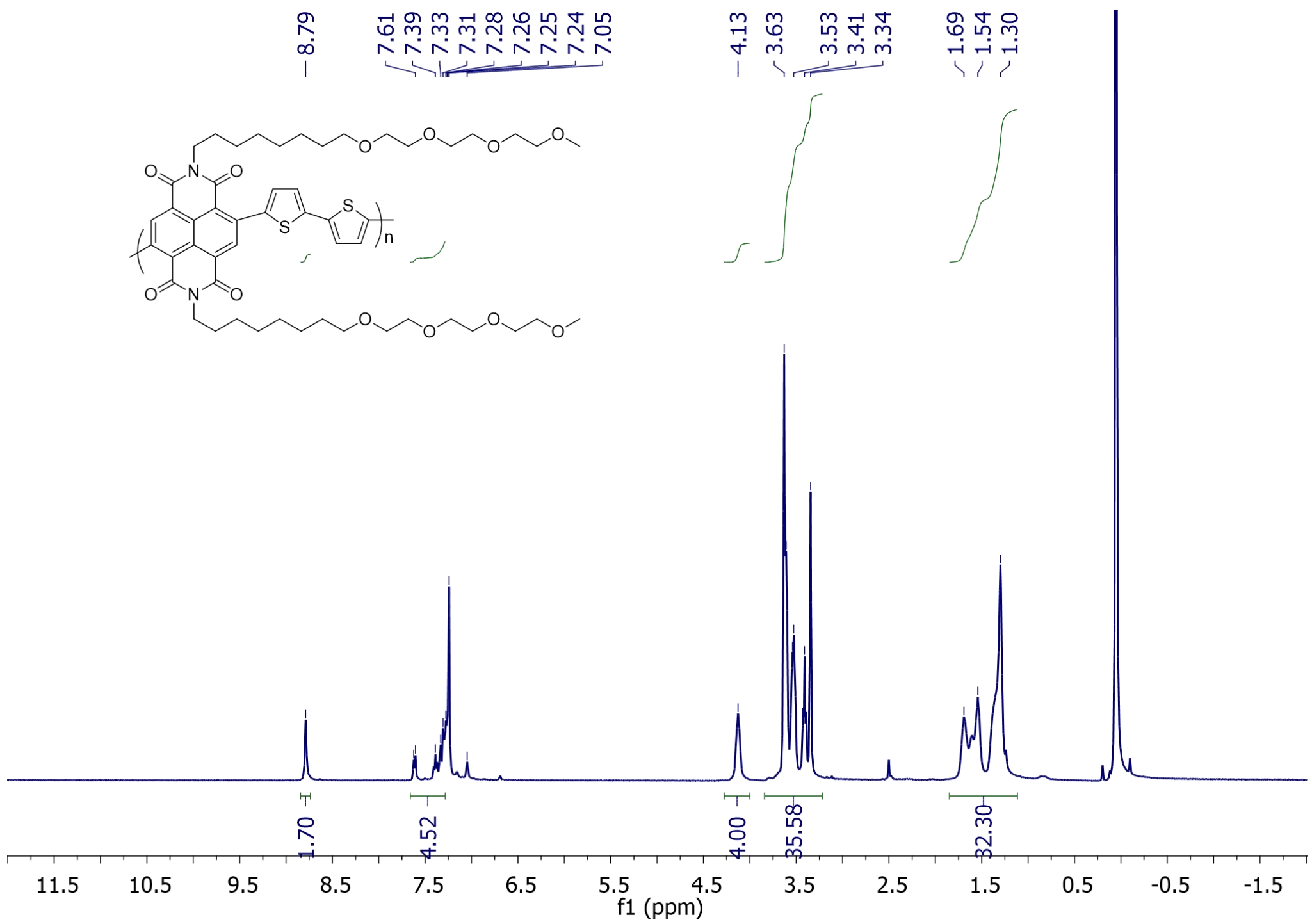

Figure S1. ^{1}HNMR spectrum of PNDI-2T.

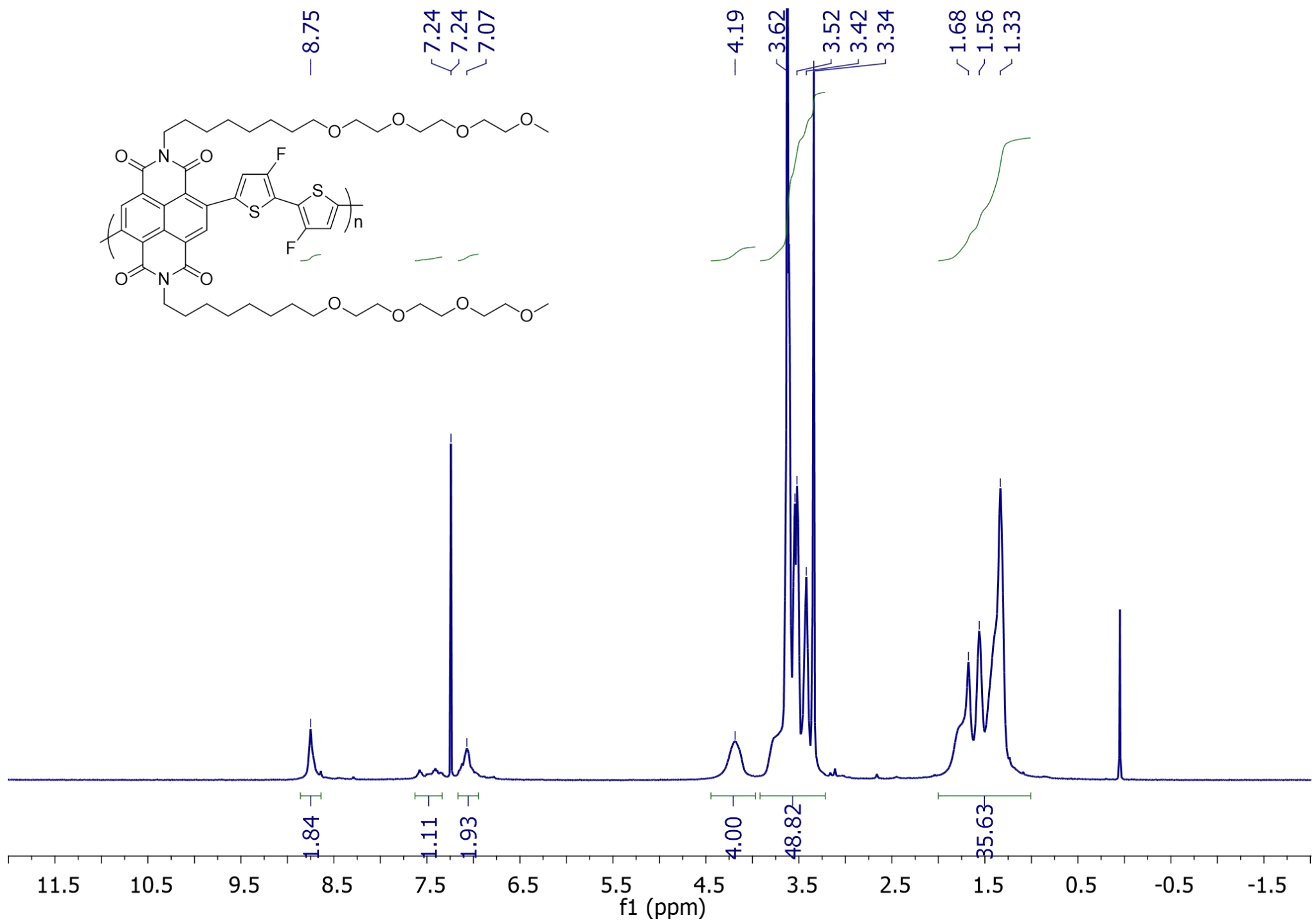

Figure S2. ¹HNMR spectrum of PNDI-2TF.

3. IR Spectra of NDI-based copolymers

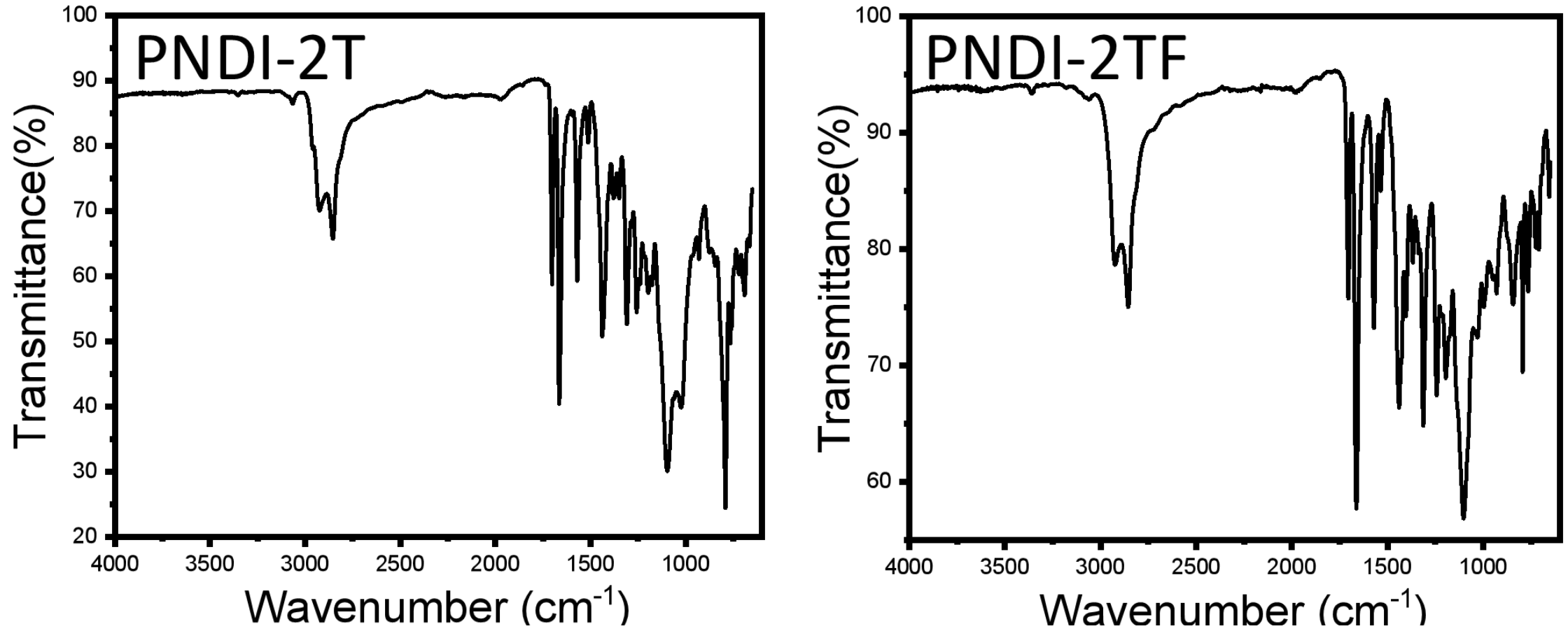

Figure S3. IR spectrum of NDI-based copolymers PNDI-2T and PNDI-2TF.

4. GPC trace of NDI-based copolymers

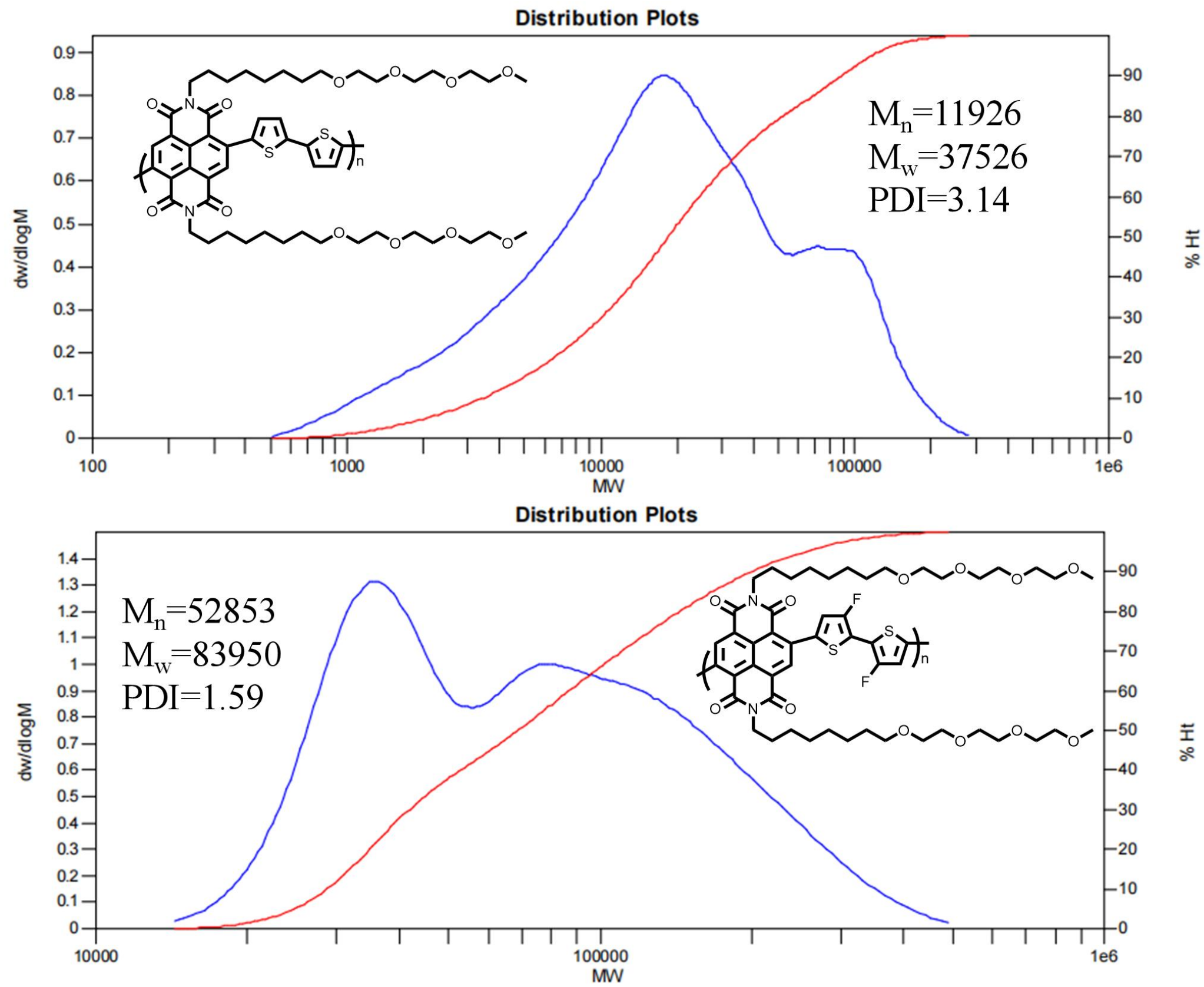

Figure S4. GPC trace of NDI-based copolymers PNDI-2T and PNDI-2TF.

5. Thermal properties of NDI-Based Copolymers

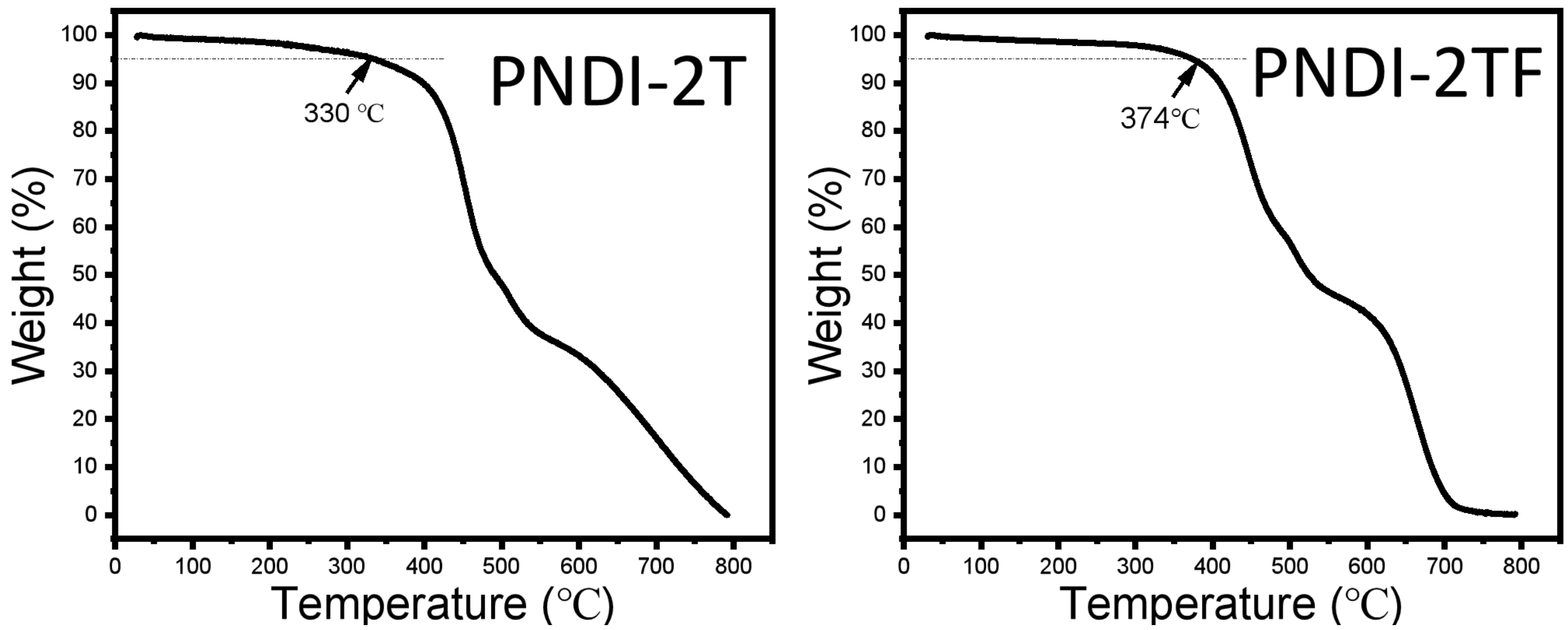

Figure S5. TGA curves of NDI-based copolymers PNDI-2T and PNDI-2TF.

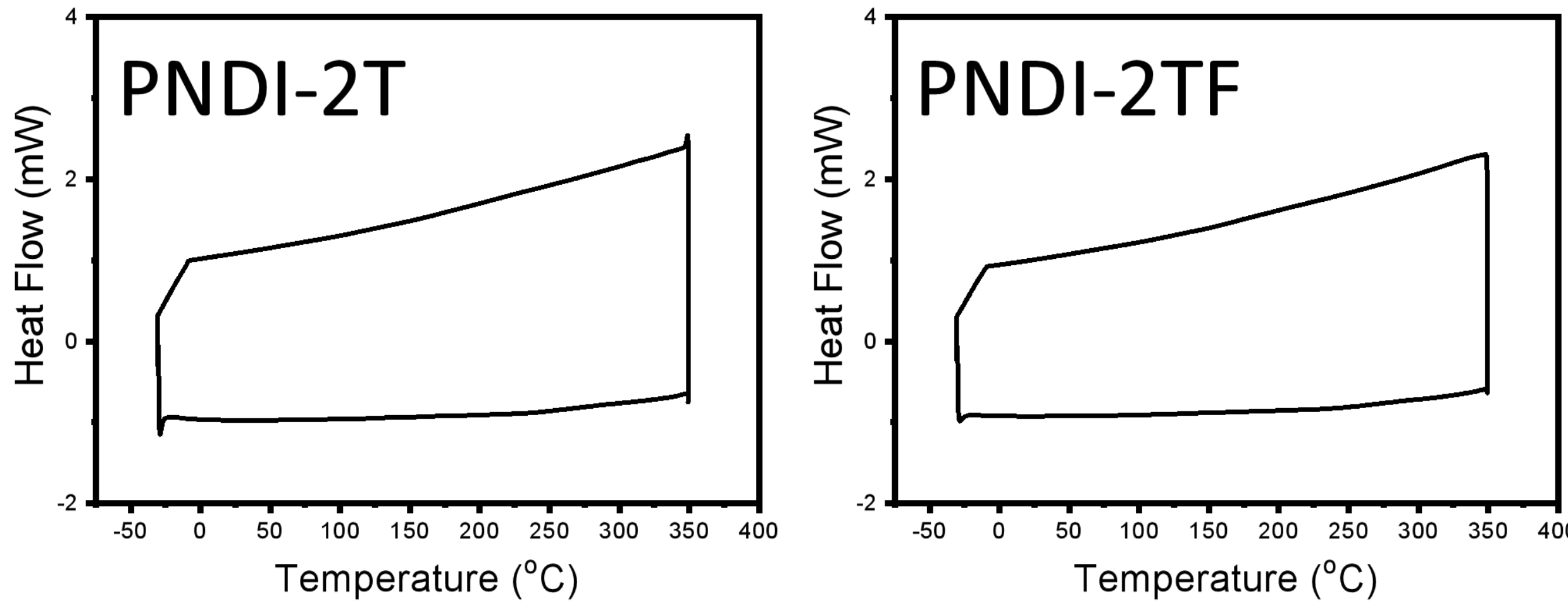

Figure S6. DSC curves of NDI-based copolymers PNDI-2T and PNDI-2TF.

6. Cyclic voltammograms of NDI-Based copolymers in CH_3CN

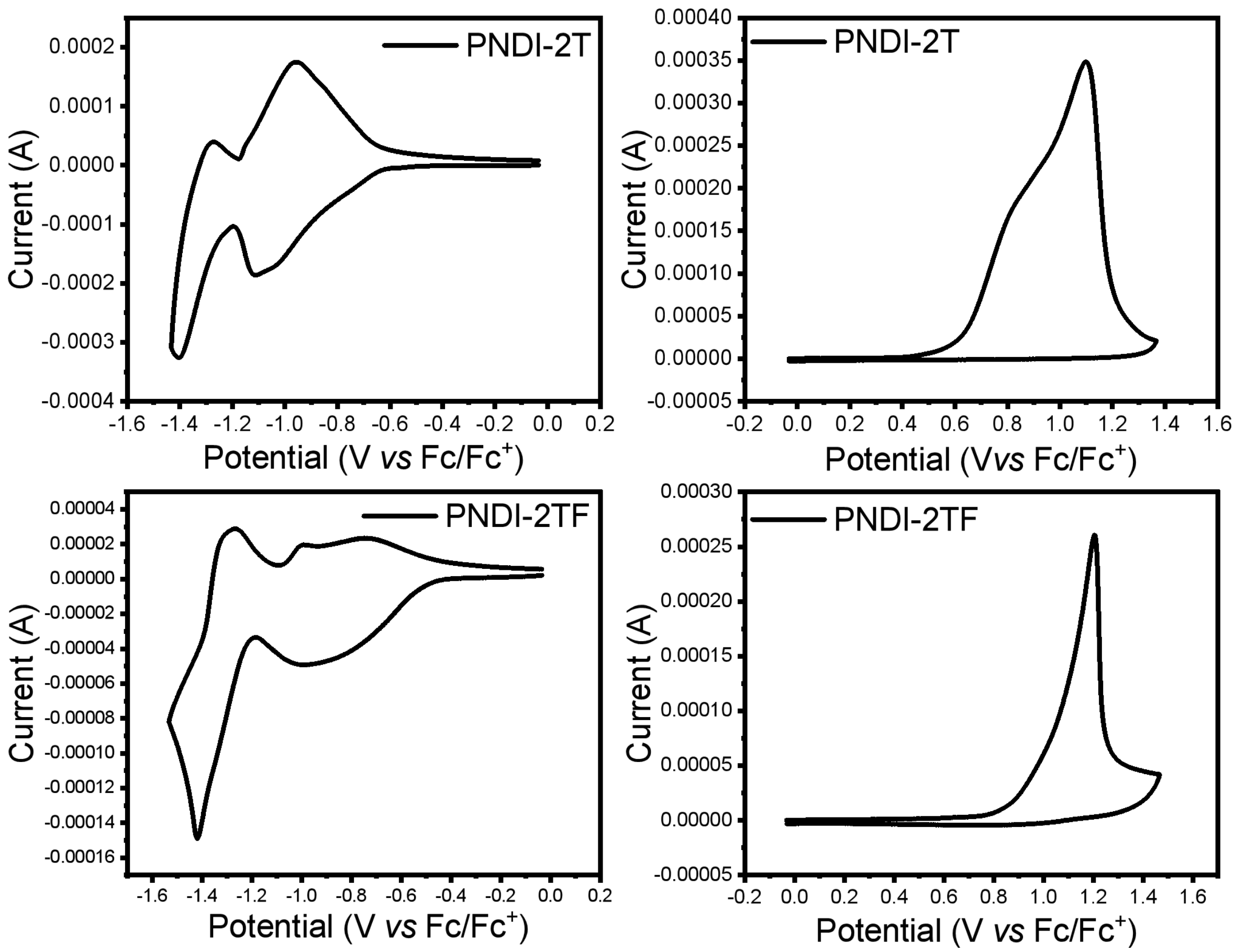

Figure S7. The cyclic voltammograms of NDI-based conjugated polymer PNDI-2T and PNDI-2TF thin films deposited on glass carbon working electrode in CH_3CN solutioncontaining Bu_4NPF_6 (0.1 mol L^{-1}) electrolyte at a scanning rate of 100 mV s^{-1}.

7. Density functional theory calculation

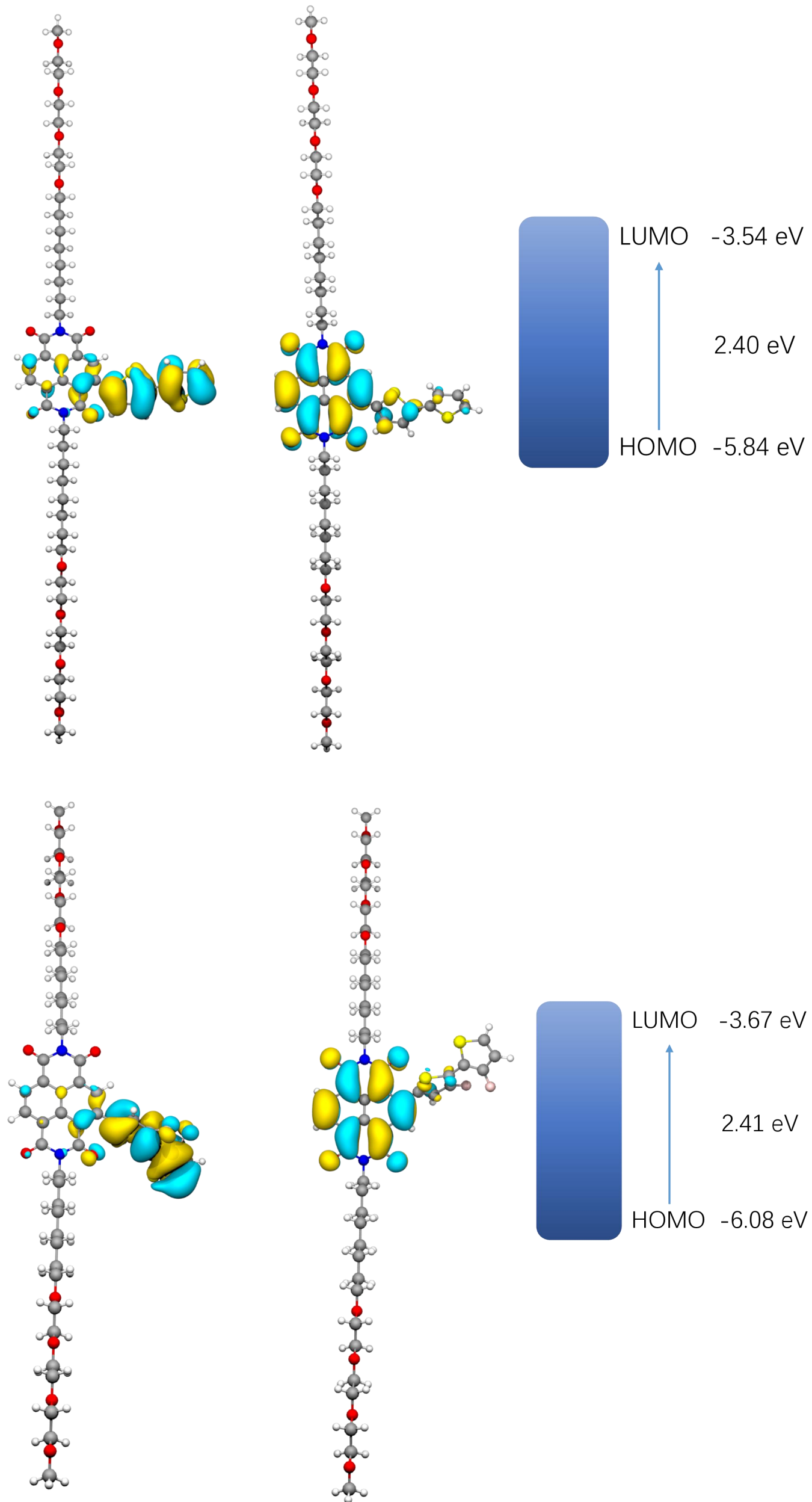

Figure S8. The calculated LUMO and HOMO for PNDI-2T (top) repeat unit and PNDI-2TF repeat unit (bottom) in gas phase.

To gain insights into the donor/acceptor character of NDI based copolymers, we carried out density functional theory (DFT) calculations at the B3LYP/6-31G(d,p) level using Gaussian 16[2]. To simplify the calculations, our model molecules contain only one repeating unit. As shown in Figure S8, all the HOMO orbitals and LUMO orbitals are isolated on their donor and acceptor moieties, respectively, in all the NDI based copolymers. All the optimized model molecules present twist structure in gas phase calculations.

8. Polymer thin film characterization

Cyclic Voltammetry (CV) in aqueous solution

Cyclic voltammetry measurements in aqueous solution were performed using a Biologic SP-150 potentiostat with a standard three electrodes configuration in 100 mM NaCl aqueous solution. Polymer thin films were spun on ITO glass using as working electrode, together with a platinum wire as the counter electrode and an Ag/AgCl electrode as the reference electrode.

Electrochemical Impedance Spectroscopy (EIS)

Electrochemical impedance spectroscopy was also carried out using a CHI 760 Evoltammetric potentiostat in a three-electrode configuration, where the working electrode was a monocrystalline silicon evaporated with 3 nm Ge, 50 nm Au, and the material spun on the Au sheet, the counter electrode was a platinum sheet, and the reference electrode was Ag/AgCl. Electrochemical impedance spectroscopy was performed on the polymer films in 0.1 M NaCl electrolyte solution at a scan rate of 50 mV s^{-1}.

UV-Vis-NIR Spectroelectrochemical measurements

Spectroelectrochemical measurements were conducted using a Shimadzu UV-1900i coupled with an electrochemical workstation. A polymer solution of 5 mg/mL was deposited on the surface of ITO through spin coating (at 1000 rpm for 30 s). The coated ITO served as the working electrode and was placed in the cuvette. An Ag/AgCl electrode was employed as reference and counter electrode, and all electrode were immersed in a 0.1 M NaCl solution. Prior to the commencement of the test, background measurements were carried out using a clean ITO substrate.

Grazing Incidence Wide Angle X-ray (GIWAXS) characterization

GIWAXS experiments were carried out on a Xeuss 3.0 UHR system from Xenocs. The instrument is equipped with a Eiger 1M detector with a pixel size of 75 µm × 75 µm. The X-ray source is a Microfocus Sealed Tube X-ray Cu-source. The wavelength used is λ = 1.54 Å. The samples were placed vertically on the goniometer at a grazing angle of 0.2° relative to the incident beam. The sample to detector distance (SDD) is 75 mm. The accumulation time for each measurement was 5 minutes.

Film Thickness measurements

The thickness of the polymer film was characterized using a Dektak XT step film thickness measurement instrument.

Atomic Force Microscope (AFM) measurements

The polymer film was characterized using an AFM (Bruker Nano Analytics). The equipment used was an Atomic Force Microscope with model Dimension FastScan, probe type FMV-A, and data analysis performed using NanoScope Analysis 3.00. The experimental procedure was as follows: An 8 µL aliquot of the prepared 5 mg/mL polymer solution was taken and spin-coated onto the glass surface, followed by annealing at 100°C for 30 min. AFM measurements were performed in contact mode with a scan size set to 3 µm, and the tests were conducted at a room temperature of approximately 25°C.

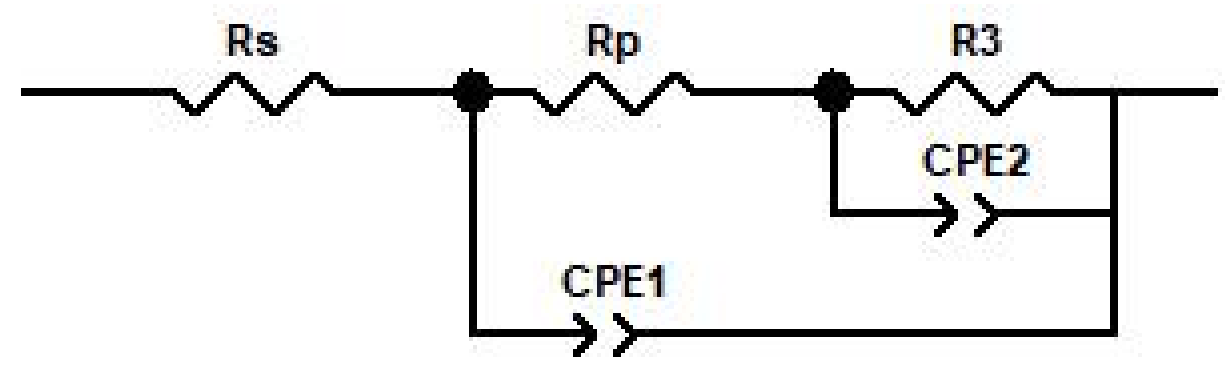

Figure S9. Equivalent fitted circuits with double capacitive elements.

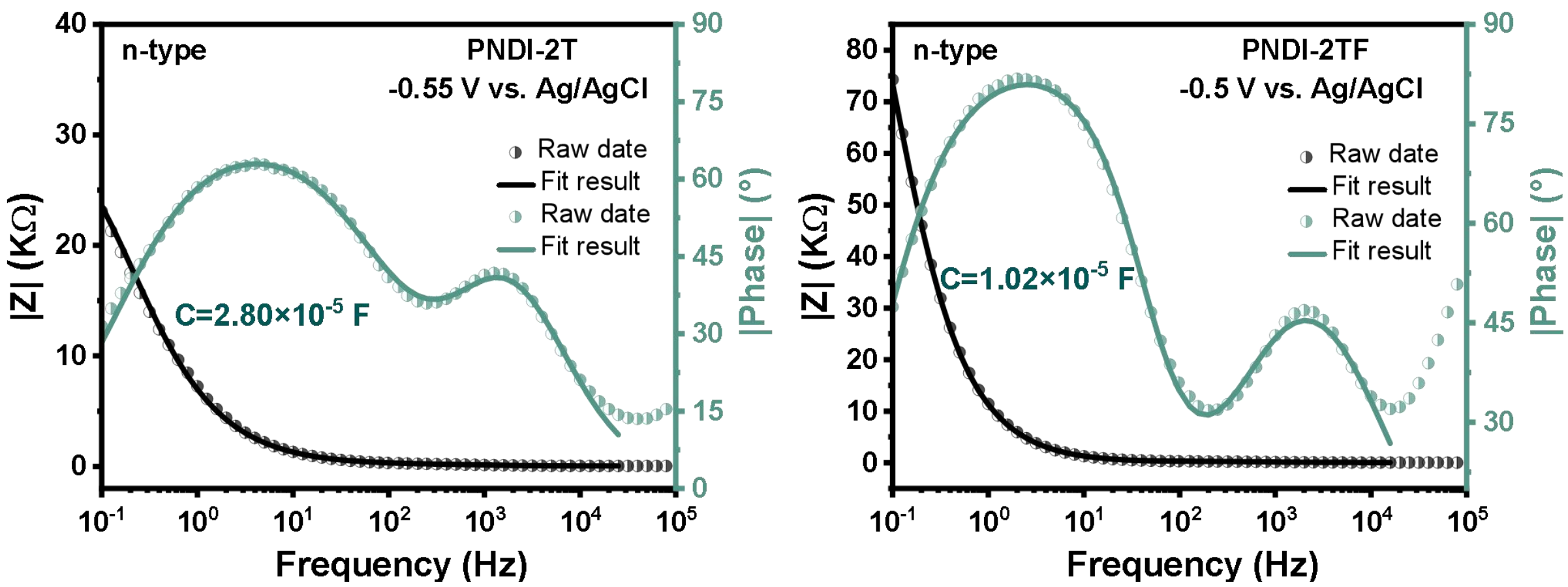

Figure S10. Bode diagrams of PNDI-2T and PNDI-2TF.

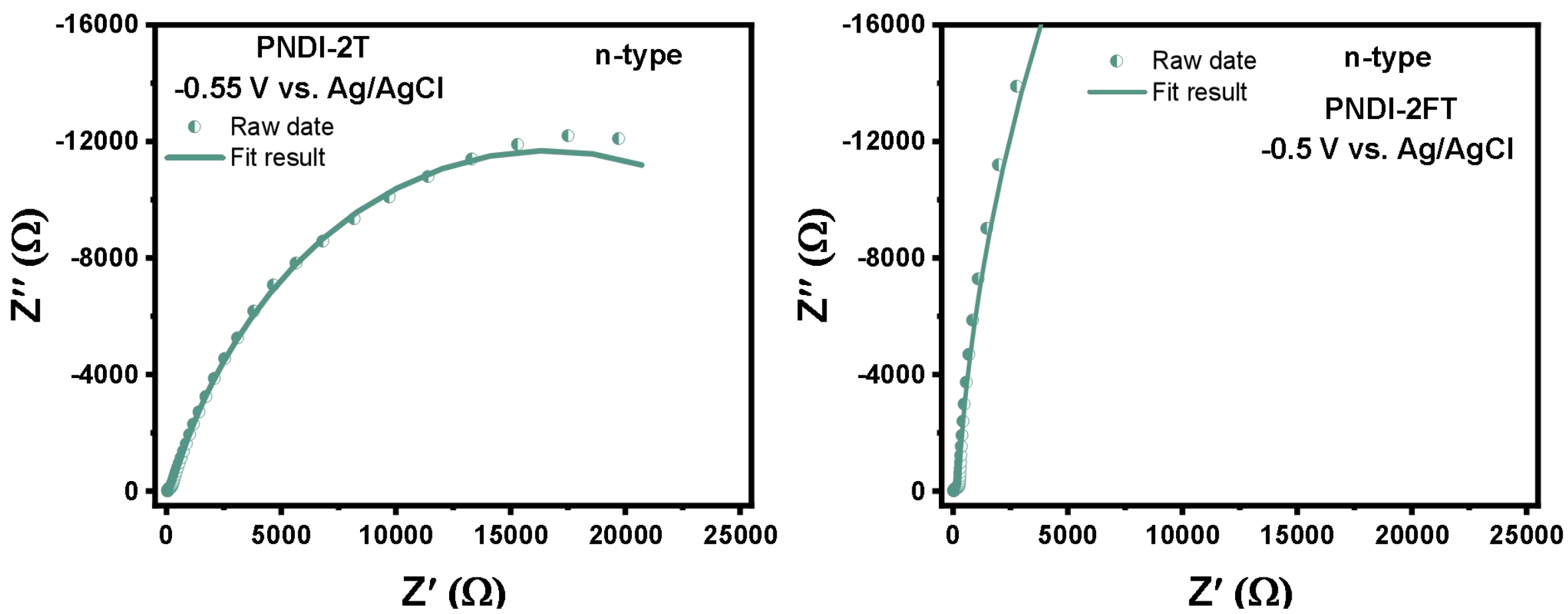

Figure S11. Nyquist diagrams of PNDI-2T and PNDI-2TF.

9. OECT devices fabrication and characterization.

The polymer solutions were prepared in chloroform (5 mg/mL). The interdigitated microelectrodes were treated with UV ozone for more than 15 min, following spin-coating the polymer solutions at 1000 rpm for 30 s. The samples were annealed on the hotplate at 100 °C for 30 mins. The electrical characterization of OECTs is recorded by a Keithley sourcemeter 2602B, which is controlled by the software Arkeo developed by Cici research.

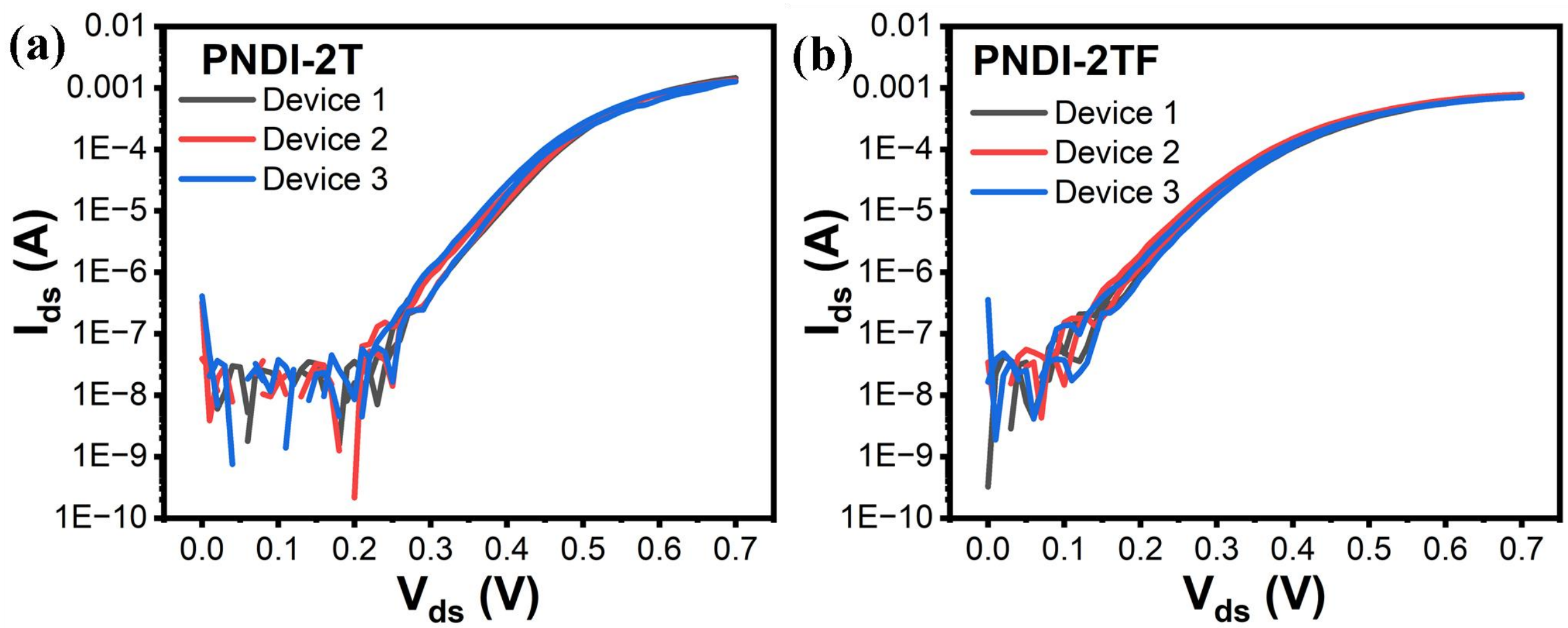

Figure S12. The transfer curves of the (a) PNDI-2T-based three OECT devices and (b) PNDI-2TF-based three OECT devices.

10. Organic field-effect transistor (OFET) fabrication and performance of polymers.

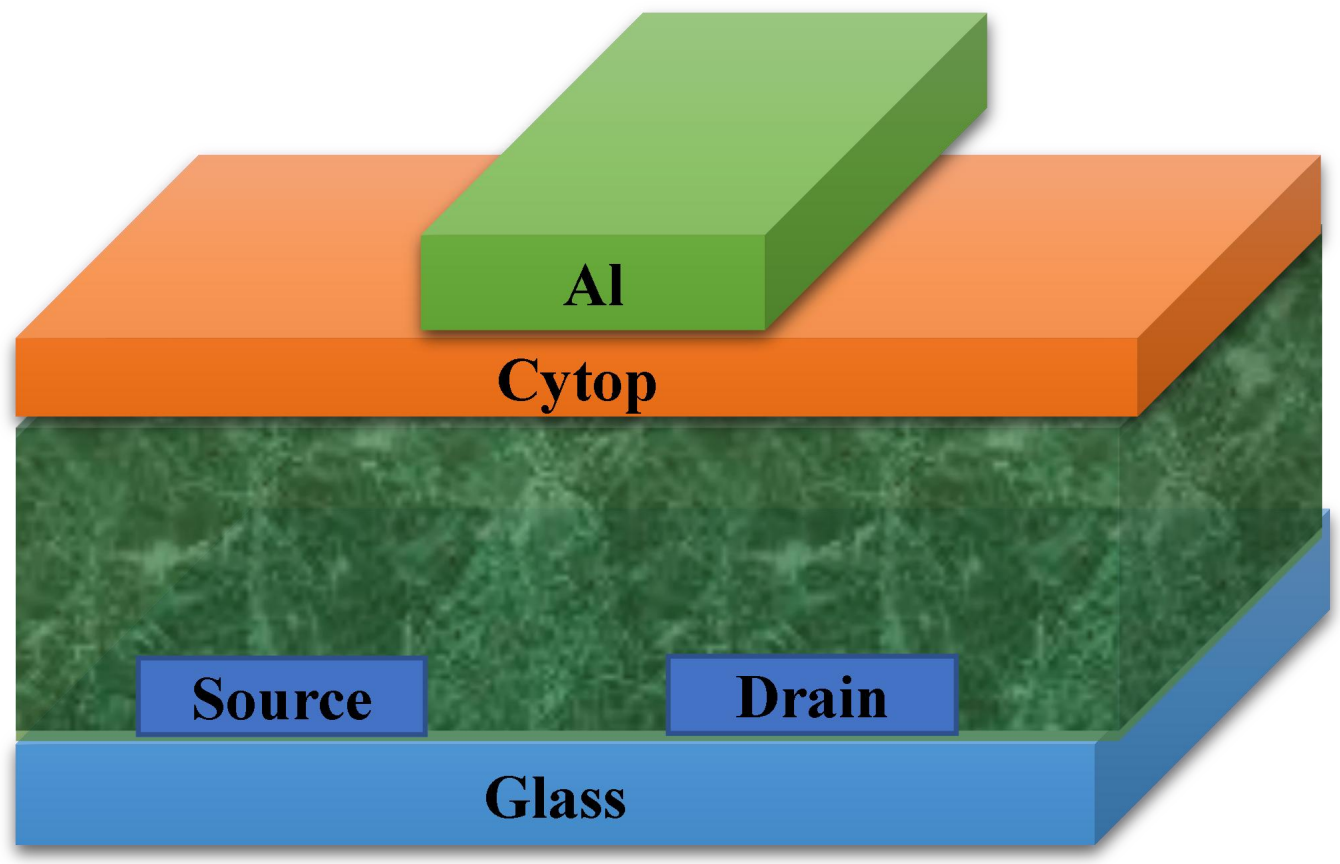

Figure S13. Schematic diagram of the OEFT device structure.

OFET fabrication and characterization. The top-gate/bottom-contact organic field-effect transistors (OFETs) were fabricated to investigate the charge transport properties of all polymers. Source/drain electrodes (3 nm Cr and 30 nm Au) were patterned on borosilicate glass by photolithography. The substrates were cleaned by sonication in acetone and isopropanol followed by UV-ozone and oxygen plasma treatment. The semiconductor layers (PNDI-2T and PNDI-2TF) were dissolved in chloroform and were spin-coated from 5 mg/mL solutions at 1500 rpm, and then they were thermally annealed at 120 °C temperatures for 10 min. The CYTOP-M material was purchased from Asahi Glass Co. Ltd., Japan. It was diluted in CT-SOLV180 solvent (volume ratio 2:1, CYTOP-M:CT-SOLV180) prior to use for dielectric coating. After spin-coating on the substrate, the dielectric layers were annealed at 100 °C for 10 min. The thickness of the dielectric layer is around 400 nm measured by profilometer and the area capacitance is calculated to be ca. 4.4 nF cm^{-2}. Finally, 50 nm Al was evaporated on top as the gate electrode to complete the devices. The channel length is 10 µm and the width is 5 mm. The devices were characterized by a Keithley 4200 semiconductor characterization system. All device fabrication and characterization were carried out in N2-filled glove box.

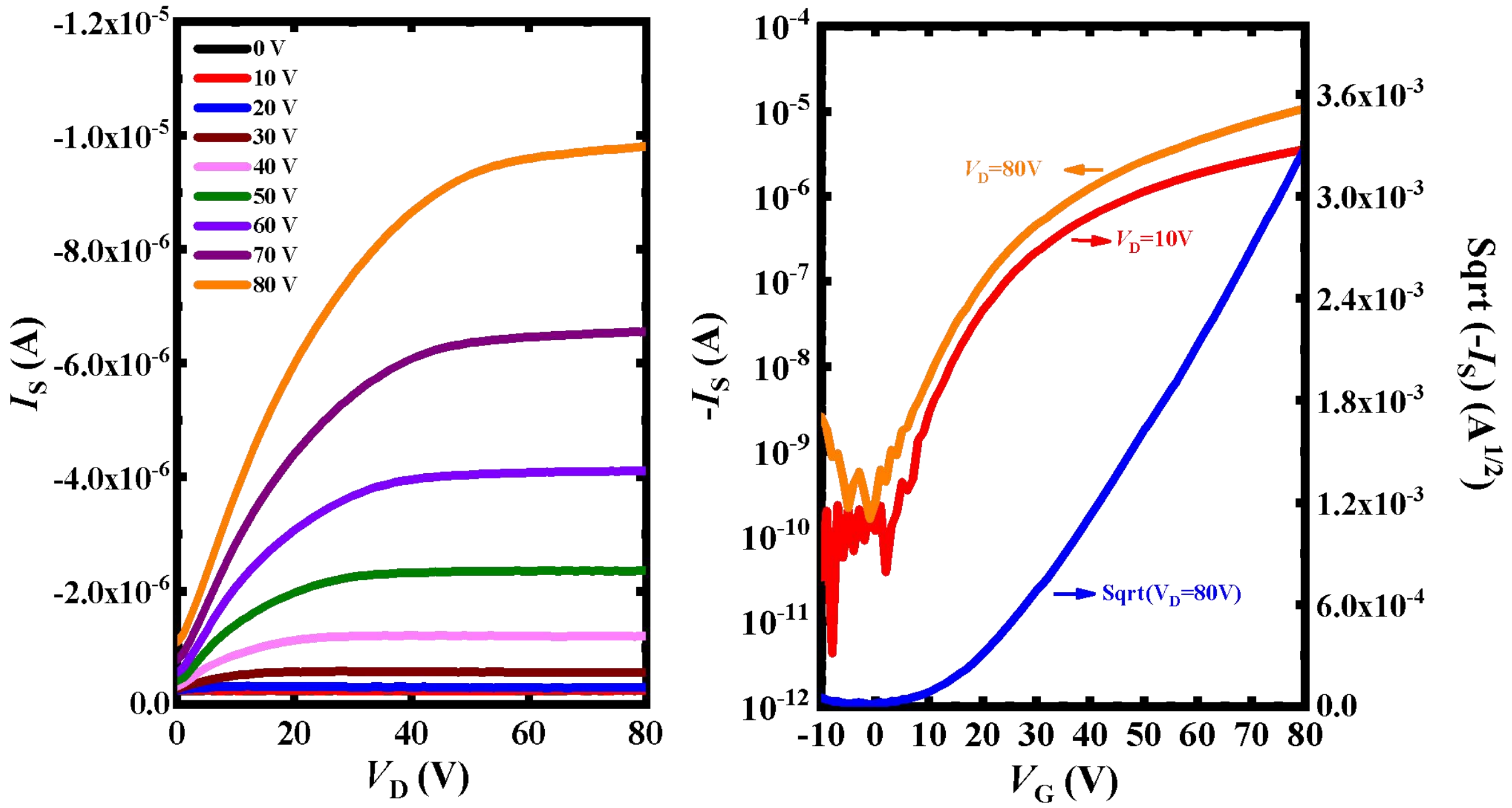

Figure S14. Output (left) and transfer (right) characteristics of top-gate/bottom-contact OFET of PNDI-2T. L = 10 μm and W = 5 mm for all devices.

11. Glucose Bio-Sensors

Electrochemical evaluation

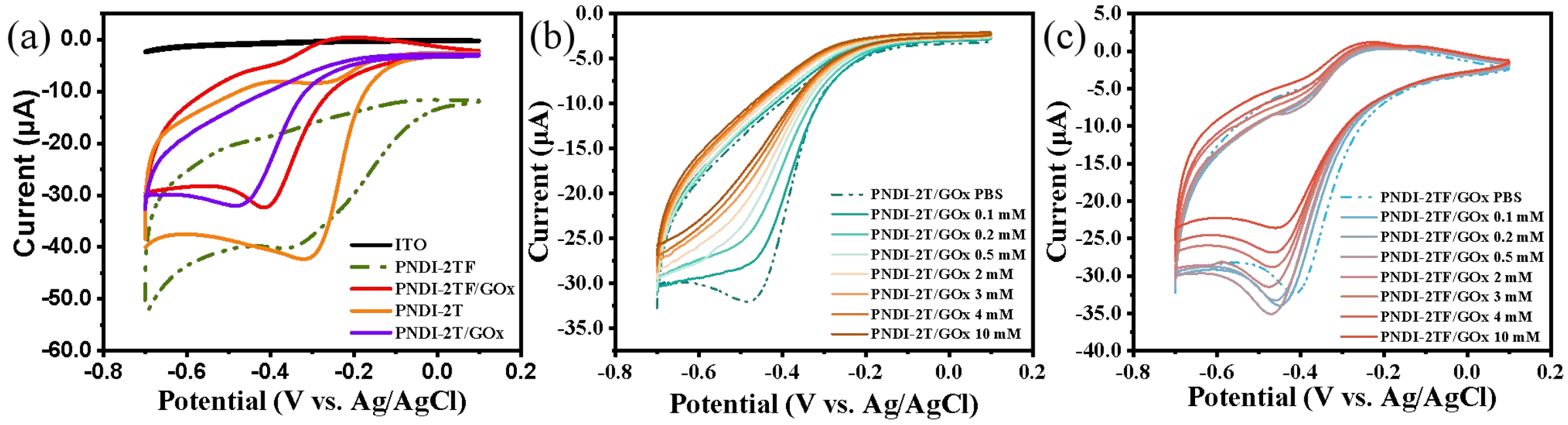

Figure 15. Electrochemical behavior of PNDI-2T and PNDI-2TF. (a) CV profiles of bare ITO, PNDI-2T/ITO, PNDI-2TF/ITO, PNDI-2T/GOx/ITO, and PNDI-2TF/GOx/ITO in PBS. (b) Glucose response of PNDI-2T/GOx-modified ITO electrodes. (c) Glucose response of PNDI-2TF/GOx-modified ITO electrodes.

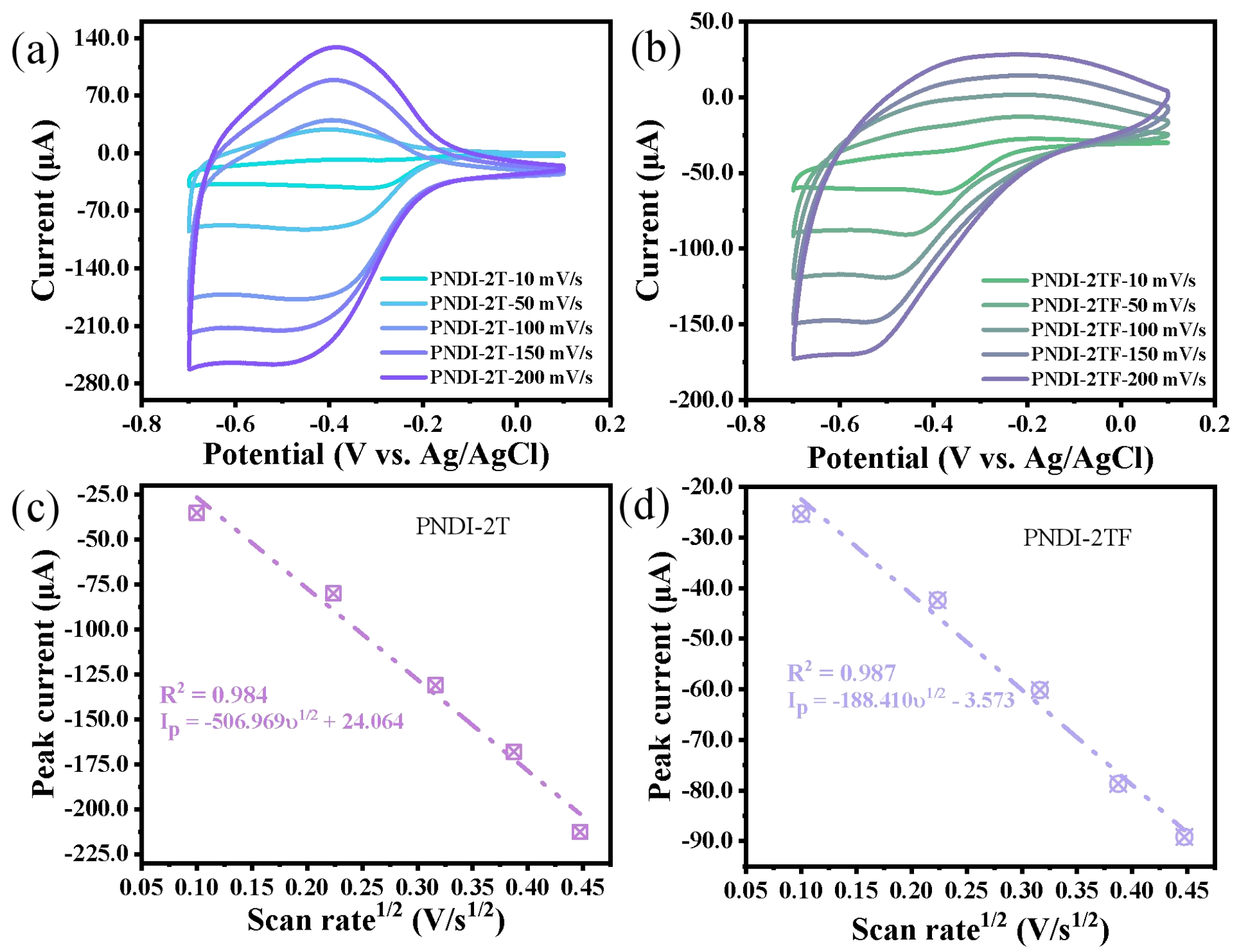

Figure S16. Scan-rate-dependent CV response. (a, b) CV curves of PNDI-2T- and PNDI-2TF-modified ITO electrodes in PBS at different scan rates. (c, d) Linear dependence of peak current on the square root of scan rate for PNDI-2T and PNDI-2TF.

All electrochemical measurements in this study were conducted using a three-electrode configuration, in which the modified ITO glass substrate (1 cm × 3 cm × 1.1 mm) served as the working electrode, a platinum plate electrode was used as the counter electrode, and a saturated Ag/AgCl electrode acted as the reference electrode. The polymer films were deposited on a 1 cm × 1 cm conductive region of the ITO substrate. Cyclic voltammetry (CV) was employed to evaluate the electrochemical behavior of PNDI-2T, PNDI-2TF, PNDI-2T/GOx, and PNDI-2TF/GOx (scan rate: 10 mV/s; potential window: −0.7 V to 0.1 V). The responses of PNDI-2T/GOx and PNDI-2TF/GOx toward glucose were qualitatively assessed by monitoring the CV variations under different glucose concentrations.

The electrochemical behavior of PNDI-2T and PNDI-2TF was evaluated to confirm their potential for glucose sensing. As shown in Figure S14a, We clearly observed the differences between the CV curves of the PNDI-2T- and PNDI-2TF-modified ITO electrodes and that of bare ITO, confirming successful modification of the ITO surface with n-type polymers. Compared with PNDI-2T/ITO and PNDI-2TF/ITO, both PNDI-2T/GOx/ITO and PNDI-2TF/GOx/ITO exhibited notable changes in CV profile-including a reduced enclosed area, decreased reduction peak intensity with a shift toward more negative potentials, and an upward shift in the baseline current. These observations indirectly verify the successful adsorption of GOx onto PNDI-2T and PNDI-2TF films. To further assess sensing capability, CV measurements were performed on the same device under glucose solutions of varying concentrations (Figure S14b,c). The CV curves of PNDI-2T/GOx/ITO and PNDI-2TF/GOx/ITO displayed pronounced changes within the

glucose concentration range of 0–10 mM, particularly in the reduction peak current. These results strongly support both the effective adsorption of GOx and the feasibility of utilizing PNDI-2T and PNDI-2TF as electrochemical glucose sensing materials. Figure S15 presents the relationship between the reduction peak currents and scan rates. The peak currents exhibit a linear dependence on the square root of the scan rate, indicating a diffusion-controlled process. This suggests that factors such as analyte concentration and diffusion barriers govern the diffusion-limited current response.

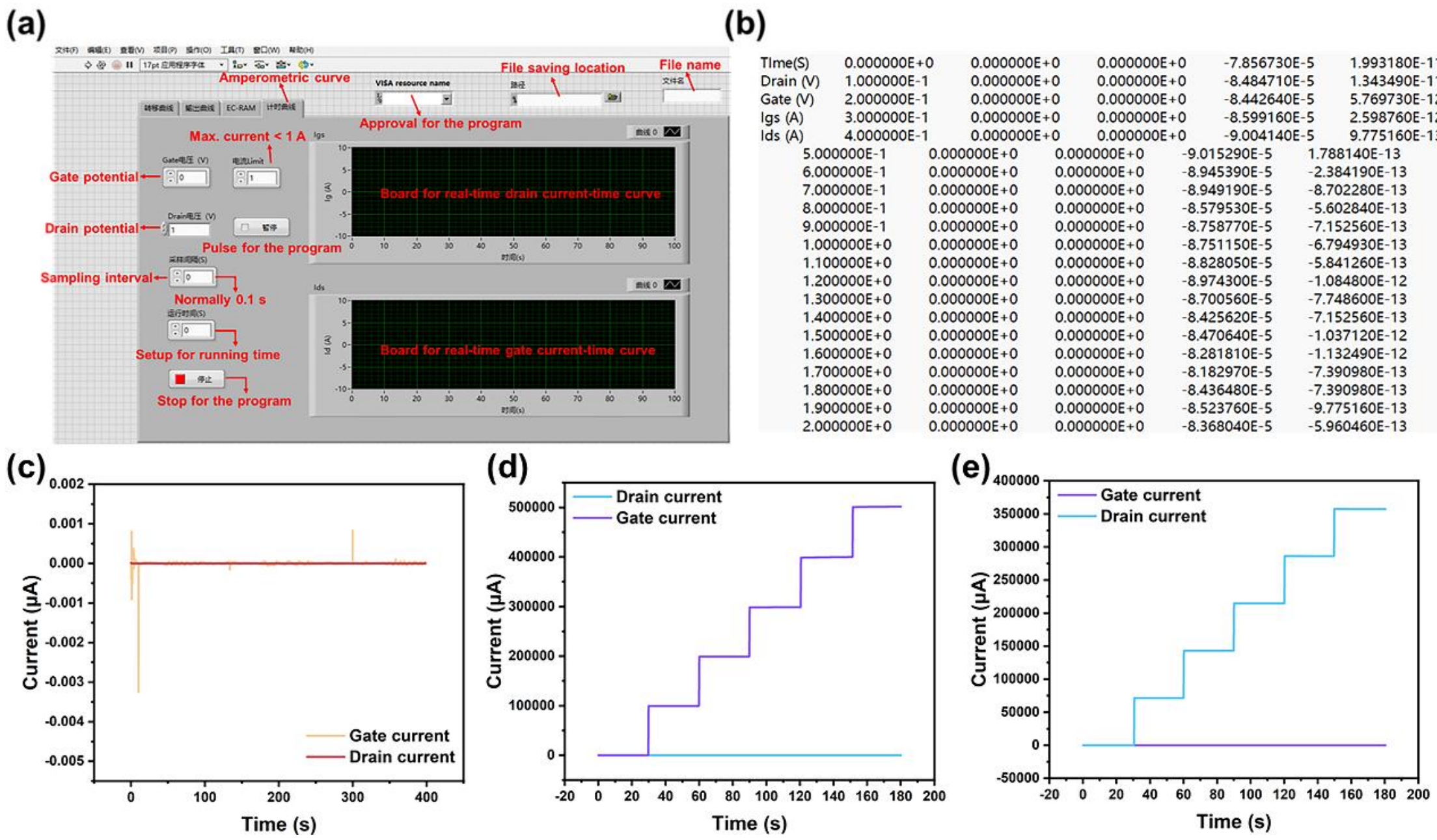

Figure S17. LabVIEW design for glucose sensing. (a) User interface for setting input parameters. (b) Text output file generated for glucose sensing measurements. (c) Current curve under open-circuit conditions. (d) Current curve with gate–source shorted and drain–source open. (e) Current curve with drain–source shorted and gate–source open.

OECT based sensor fabrication

First, PNDI-2T and PNDI-2TF were dissolved in chloroform to obtain polymer solutions with a concentration of 5 mg mL^{-1}. An appropriate amount of each solution was then rapidly dispensed onto the conductive surface of the ITO glass or onto the channel region of the interdigitated electrodes. The films were subsequently spin-coated at 1000 rpm for 30 s, followed by thermal annealing for 30 min. The fabricated devices were stored in a desiccator at room temperature prior to use. For devices requiring further functionalization with glucose oxidase (GOx), a 10 mg mL^{-1} GOx solution was drop-cast onto the polymer film and allowed to undergo physical adsorption for at least 30 min. After adsorption, any excess GOx solution was gently wicked away using a dust-free cloth, ensuring that the wipe did not contact the device surface to prevent removal of the immobilized enzyme. Devices modified with GOx were measured on the same day to ensure optimal enzymatic activity. The source meter (Keithley 2612B) was employed for the electrical characterization of the devices.

Glucose sensing

The glucose sensing measurements were conducted using a custom-designed LabVIEW program. Figure S16 illustrates the overall program interface and the validation of its functionality. Users can manually input the required sensing parameters (Figure S16a) and obtain the corresponding operational data outputs (Figure S16b). Subsequent electrical verification (Figure S16c-e) shows that the current remains zero under open-circuit conditions and increases linearly with voltage under short-circuit conditions, consistent with Ohm’s law. These results confirm the reliability and usability of the program.

For n-type polymer OECT based sensor, firstly, 20 μL of PBS was carefully drop-cast onto the GOx-modified device. Secondly, after an initial stabilization period, the program was paused, and the PBS was gently removed using a dust-free cloth. Thirdly, 20 μL of PBS was applied onto the device, and the program was resumed to record the current response for 50 s. Fourthly, the PBS was removed, and a glucose solution of a defined concentration was drop-cast onto the device. The program was restarted to collect the current response for another 50 s. In the fifth step, Step 4 was repeated with glucose solutions of increasing concentrations to evaluate the glucose sensing performance of the device. The glucose response was quantified using two approaches: (i) the absolute current change (ΔI_D), and (ii) the normalized response (*NR*).

$$\Delta I_D = I_{D1} - I_0$$

$$NR = \left|\frac{I_{D1} - I_0}{I_0}\right| \times 100\%$$

where I_{D1} is the drain current measured at a specific glucose concentration, and I_0 is the drain current recorded in the absence of glucose.

Contact angle and zeta potential measurements

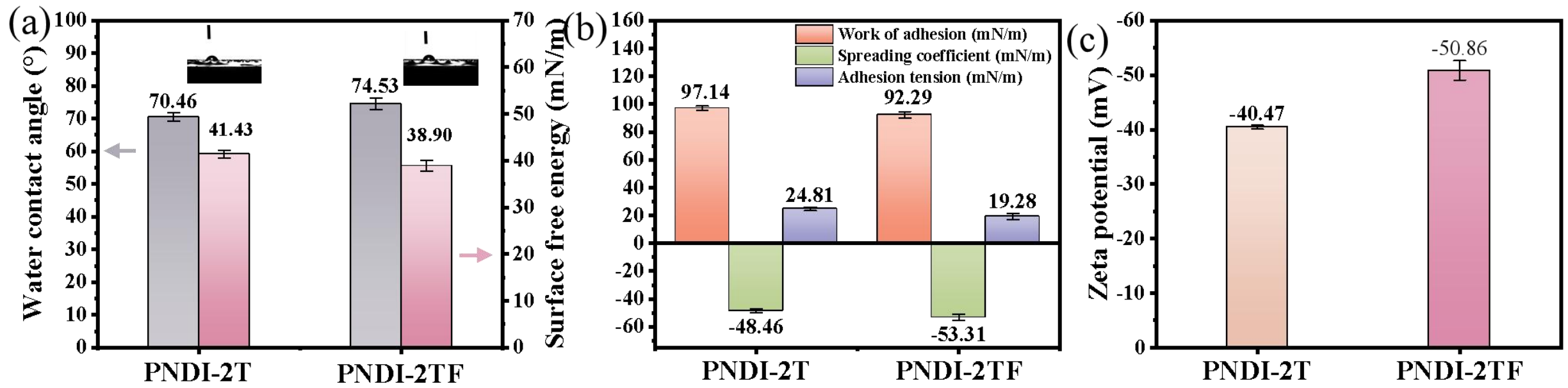

Figure S18. Surface properties of PNDI-2T and PNDI-2TF films. (a, b) Surface wettability of PNDI-2T and PNDI-2TF films. (c) Zeta potential of PNDI-2T and PNDI-2TF films.

The surface hydrophilicity of PNDI-2T and PNDI-2TF films was evaluated using a contact angle goniometer (CA100). Each polymer was spin-coated onto ITO glass substrates to form thin films for the measurements. Contact angle determination was performed following the ASTM D5946-4 standard. Briefly, a 2 μL water droplet was dispensed onto the film surface, and the contact angle was recorded after a stabilization period of 60 s. For each film, five random locations were selected on each side of the coated area, and the reported contact angle was calculated as the average of the ten measured points. The zeta potential of the films was measured using the streaming potential method. The n-type polymers films were prepared by spin-coating onto quartz glass slides (2 cm × 1 cm × 1 mm). The treated slides were mounted in an Anton Paar SurPASS 3 solid-surface zeta potential analyzer and measured in 1 mM KCl solution (pH = 7).

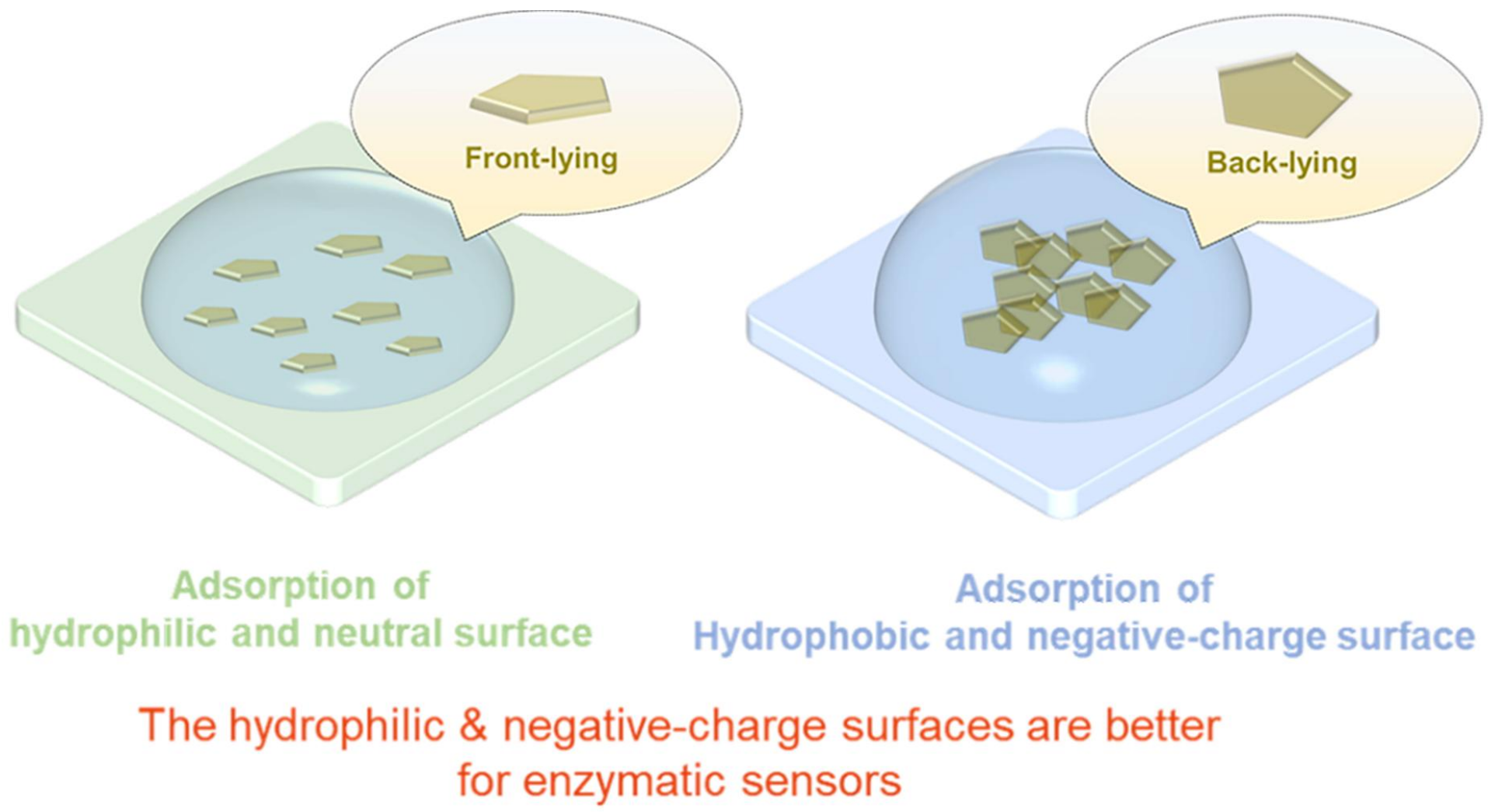

Figure S19. Proposed enzymes adopt a "front-facing" orientation in PNDI-2T/GOx (left) and a “back-facing” in PNDI-2TF/GOx (right).

12. References

(1) Kim, R.; Kang, B.; Sin, D. H.; Choi, H. H.; Kwon, S. K.; Kim, Y. H.; Cho, K. Oligo(Ethylene Glycol)-Incorporated Hybrid Linear Alkyl Side Chains for n-Channel Polymer Semiconductors and Their Effect on the Thin-Film Crystalline Structure. *Chem. Commun.* **2015**, *51* (8), 1524–1527.

(2) Frisch, M. J.; Trucks, G. W.; Schlegel, H. B.; Scuseria, G. E.; Robb, M. a.; Cheeseman, J. R.; Scalmani, G.; Barone, V.; Petersson, G. a.; Nakatsuji, H.; et al. G16_C01. 2016, p Gaussian 16, Revision C.01, Gaussian, Inc., Wallin.
